# PPM1G Inhibits Epithelial–Mesenchymal Transition in Cholangiocarcinoma by Catalyzing TET1 Dephosphorylation for Destabilization to Impair Its Targeted Demethylation of the CLDN3 Promoter

**DOI:** 10.1002/advs.202407323

**Published:** 2024-10-30

**Authors:** Wenzheng Liu, Yiyang Kuai, Da Wang, Junsheng Chen, Fei Xiong, Guanhua Wu, Qi Wang, Wenhua Huang, Yongqiang Qi, Bing Wang, Yongjun Chen

**Affiliations:** ^1^ Department of Biliary‐Pancreatic Surgery Tongji Hospital Tongji Medical College Huazhong University of Science and Technology 1095 Jiefang Avenue Wuhan Hubei 430030 China; ^2^ Department of General Surgery Beijing Friendship Hospital Capital Medical University Beijing 100050 China; ^3^ Department of Emergency Tongji Hospital Tongji Medical College Huazhong University of Science and Technology 1095 Jiefang Avenue Wuhan Hubei 430074 China; ^4^ Key Laboratory of Laparoscopic Technology of Zhejiang Province Department of General Surgery Sir Run‐Run Shaw Hospital Zhejiang University School of Medicine Hangzhou 310016 China

**Keywords:** cholangiocarcinoma, demethylation, dephosphorylation, PPM1G, TET1

## Abstract

Ten–eleven translocation protein 1 (TET1) functions as an epigenetic regulatory molecule, mediating the majority of DNA demethylation, and plays a role in the development of different types of cancers by regulating the expression of proto‐oncogenes and oncogenes. Here it is found that TET1 is highly expressed in cholangiocarcinoma (CCA) and is associated with a poor prognosis. In addition, TET1 promotes claudin‐3 (CLDN3) transcription by targeting the CLDN3 promoter region between −16 and 512 for demethylation. PPM1G functions as a protein dephosphorylase, catalyzing the dephosphorylation of TET1. This results in the destabilization of the TET1 protein, thereby impairing the targeting of the CLDN3 promoter for demethylation. Two phosphatase inhibitors, staurosporine and AZD0156, inhibit epithelial‐to‐mesenchymal transition (EMT) in cholangiocarcinoma cells by suppressing TET1 expression. In conclusion, it is also demonstrated that PPM1G can be employed as a therapeutic target to impede the progression of CCA by catalyzing the dephosphorylation of TET1, which diminishes the capacity of TET1 to target the CLDN3 promoter to activate transcription and inhibit EMT in CCA.

## Graphical abstract

Ten–eleven translocation protein 1 (TET1) exerts its pro‐carcinogenic effect in cholangiocarcinoma by catalyzing the demethylation of the claudin‐3 (CLDN3) promoter region. This effect is impaired by dephosphorylation of TET1 by PPM1G, destabilizing the TET1 protein. In addition, two phosphorylation inhibitors, staurosporine and AZD0156, inhibit cholangiocarcinoma progression by destabilizing the protein by inhibiting TET1 phosphorylation.

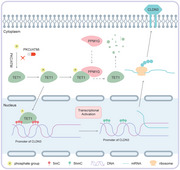

## 1 Introduction

Cholangiocarcinoma (CCA) is a prevalent primary malignant neoplasm that arises from the epithelial cells of the bile ducts. It is the second most common primary malignant tumor of the liver after hepatocellular carcinoma.^[^ ^1^ ^]^ Epidemiological data indicate that the global incidence of cholangiocarcinoma has been gradually increasing over the past decade, with a poor prognosis.^[^ ^2^ ^]^ A statistical analysis conducted by the National Comprehensive Cancer Network of the United States of America indicates that the 5 year overall survival rate for patients diagnosed with hepatoportal cholangiocarcinoma ranges from 20% to 42%. In comparison, the rate for those diagnosed with distal cholangiocarcinoma ranges from 16% to 52%.^[^ ^3^ ^]^ The optimal treatment for cholangiocarcinoma is radical surgical resection. However, only 20–30% of patients diagnosed with cholangiocarcinoma are eligible for surgical resection.^[^ ^4^ ^]^ Although combined radiotherapy can potentially delay disease progression in patients who cannot undergo surgical resection, the effect remains limited.^[^ ^5^ ^]^ The lack of specific molecular markers or targets for diagnosing and treating cholangiocarcinoma represents a significant challenge in medical research.

Epigenetics is defined as a heritable change in gene function that occurs without a change in the DNA sequence of the gene, ultimately resulting in a change in phenotype.^[^ ^6^ ^]^ The number of studies investigating the potential of epigenetic modifications to treat cancer has grown in recent years.^[^ ^7^ ^]^ However, the use of epigenetic‐related molecules as a means of inhibiting cholangiocarcinoma remains an unclear area.^[^ ^8^ ^]^ The methylation and DNA demethylation processes represent pivotal components of epigenetic inheritance.^[^ ^9^ ^]^ The majority of solid tumor cells exhibit a global reduction in DNA methylation.^[^ ^10^ ^]^ Ten–eleven translocation protein 1 (TET1), the most prevalent DNA demethylation enzyme, was identified as a fusion protein by Ono et al. during their investigation of a specific case of acute leukemia.^[^ ^11^ ^]^ TET1 can recognize and bind to specific regions of the genome that exhibit a high density of 5′‐cytosine‐phophate‐guanosine (CpG)‐3′ dinucleotides. These regions, which are often referred to as CpG islands, serve as a substrate for TET1, which catalyzes the hydroxylation of 5‐methylcytosine (5mC) to 5‐hydroxymethylcytosine (5hmC).^[^ ^12^ ^]^ This process is known as active or passive DNA demethylation, which plays a crucial role in maintaining the equilibrium between methylation and demethylation of DNA.

Additionally, it is essential for maintaining the methylation homeostasis of the genome, which is vital to achieving epigenetic regulation.^[^ ^13^ ^]^ TET1 displays considerable heterogeneity across different tumor types, exhibiting oncogenic or tumor‐suppressive functions.^[^ ^14^ ^]^ Although numerous studies have examined the function of TET1 in CCA, the potential for exploiting the epigenetic capabilities of TET1 to impede CCA progression remains largely uninvestigated territory. By transcriptome sequencing (RNA sequencing, RNA‐Seq) and bisulfite post‐transformation polymerase chain reaction (BSP), we demonstrated that TET1 relies on its catalytic activity to target the claudin‐3 (CLDN3) promoter region for demethylation, thereby promoting its transcription and consequently promoting epithelial‐to‐mesenchymal transition (EMT) in CCA. This was demonstrated by the characterization of CLDN3 promoter function and the targeting of demethylation.

Post‐translational modification of proteins represents an additional significant aspect of epigenetics.^[^ ^15^ ^]^ They influence biological functions by affecting the intracellular localization and stability of proteins.^[^ ^16^ ^]^ As evidenced in the literature, phosphorylation of TET1 has been demonstrated to render TET1 less susceptible to degradation.^[^ ^17^ ^]^ By high‐performance liquid chromatography‐mass spectrometry (HPLC‐MS/MS), we discovered that TET1 binds to the dephosphorylase PPM1G and is catalytically dephosphorylated, which renders the TET1 protein unstable and susceptible to degradation, thereby inhibiting the function of TET1 as an oncogene. In addition, we discovered that two distinct classes of phosphatase inhibitors can achieve this outcome.

## 2 Results

### 2.1 TET1 Is Highly Expressed in Cholangiocarcinoma and Correlates with Prognosis Concerning the Promotion of Cholangiocarcinoma Cell Proliferation, Invasion, and Migration In Vivo and In Vitro

**Figure 1:**
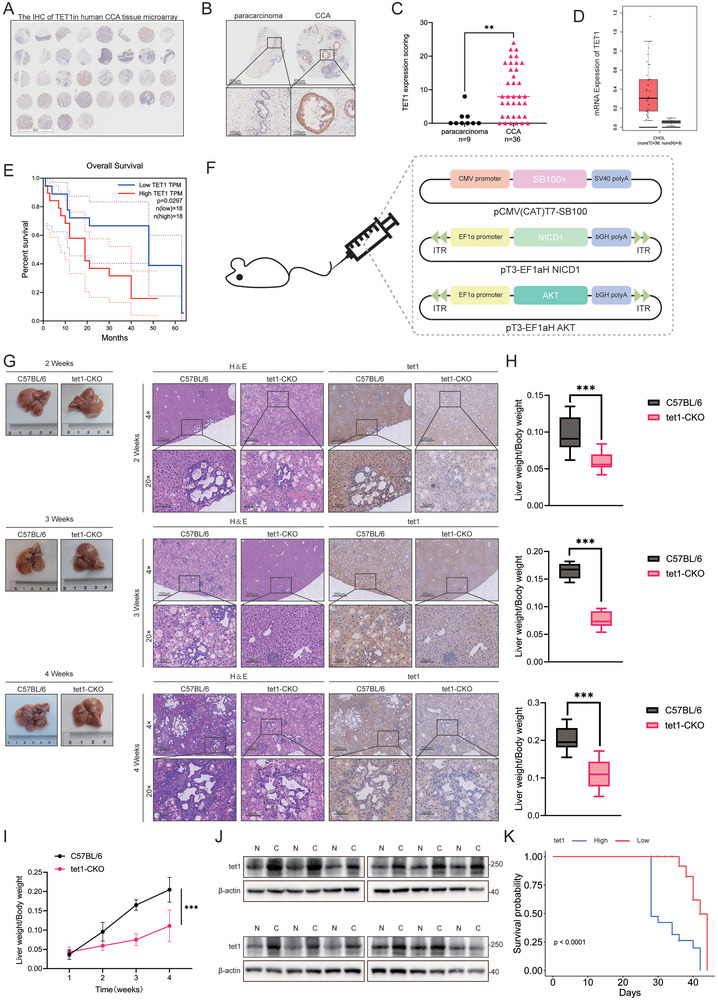
TET1 is highly expressed in CCA and is associated with prognosis. A) Immunohistochemical staining of human CCA tissue microarrays for TET1 (*n* = 36). B) Enlargement of representative CCA and normal tissue in panel (A). C) TET1 expression score based on CCA tissue microarray in panel (A). Data are expressed as mean ± standard deviation (SD). *p*‐values were calculated by unpaired, two‐tailed Student's *t*‐test. D) The expression level of TET1 in CCA from the TCGA database and corresponding normal tissues from the GTEx database. E) Following the classification of CCA patients based on the median expression of TET1 (high‐ and low‐expressing tumors; *n* = 36), Kaplan–Meier analysis was employed to estimate the overall survival of CCA patients. F) A graphical depiction of the plasmids utilized for the construction of in situ cholangiocarcinoma model mice. G) Overview, H&E staining, and TET1 IHC staining of in situ CCA models constructed in C57BL/6 mice as well as tet1‐CKO mice at 2, 3, and 4 weeks. H,I) Liver weight‐to‐body weight ratios at 2, 3, and 4 weeks in wild‐type versus tet1‐CKO mouse models of CCA in situ (*n* = 5). J) 12 pairs of cancerous versus normal tissue TET1 protein expression in in situ CCA model mice. K) Survival curves of in situ CCA models in wild‐type and tet1‐KO mice. **p < 0.01 and ***p < 0.001.

A considerable number of studies have been conducted to investigate the expression of TET1 in various cancers.^[^ ^18^ ^]^ The results of these studies have demonstrated that there are discrepancies in the expression of TET1 in different types of tumors, with varying effects on patient prognosis.^[^ ^19^ ^]^ To investigate the expression of TET1 in CCA, an immunohistochemical (IHC) analysis was performed on an extrahepatic CCA (ECCA) tissue microarray (TMA) consisting of 36 CCA tissues pathologically confirmed and 9 normal tissues. These tissues were obtained from the National Engineering Center for Biochip (Outdo Biotech, Shanghai, China). The results demonstrated a notable elevation in TET1 expression in ECCA tissues relative to paracancerous and normal bile duct tissues (*p* < 0.001) (**Figure** **1**A–C; Table S1, Supporting Information). A comparative analysis of the RNA expression data for CCA in The Cancer Genome Atlas (TCGA) database and the corresponding normal tissues from the Genotype‐Tissue Expression (GTEx) database revealed that TET1 was markedly overexpressed in CCA (Figure 1D). A Kaplan–Meier survival analysis of the TMA showed that the overall survival rate of CCA patients with elevated TET1 messenger RNA (mRNA) expression was markedly inferior to that of the low TET1 expression group (*p* < 0.1) (Figure 1E).

Subsequently, we constructed in situ CCA models in C57BL/6 mice as well as TET1 conditional knockout mice (tet1‐CKO) by hydrodynamic tail vein injection and the Sleeping Beauty transposon^[^ ^20^ ^]^ (Figure 1F) to examine the differences between cancerous and normal tissues in terms of TET1 expression, as well as the effects of TET1 on CCA progression and survival in vivo. Histological analyses employing hematoxylin–eosin (H&E) staining and IHC staining demonstrated that CCA exhibited relatively slow progression in tet1‐CKO mice (Figure 1G). The data pertaining to the liver weight‐to‐body weight ratio suggest that tet1‐CKO mice exhibit a relatively low liver weight‐to‐body weight ratio and demonstrate a relatively sluggish growth rate (Figure 1H,I). The results of western blot (WB) experiments demonstrated that tet1 expression was elevated in 12 pairs of CCA tissues compared to normal tissues (Figure 1J). Kaplan–Meier survival analyses revealed tet1‐CKO mice exhibited a relatively prolonged survival period (Figure 1K).

**Figure 2:**
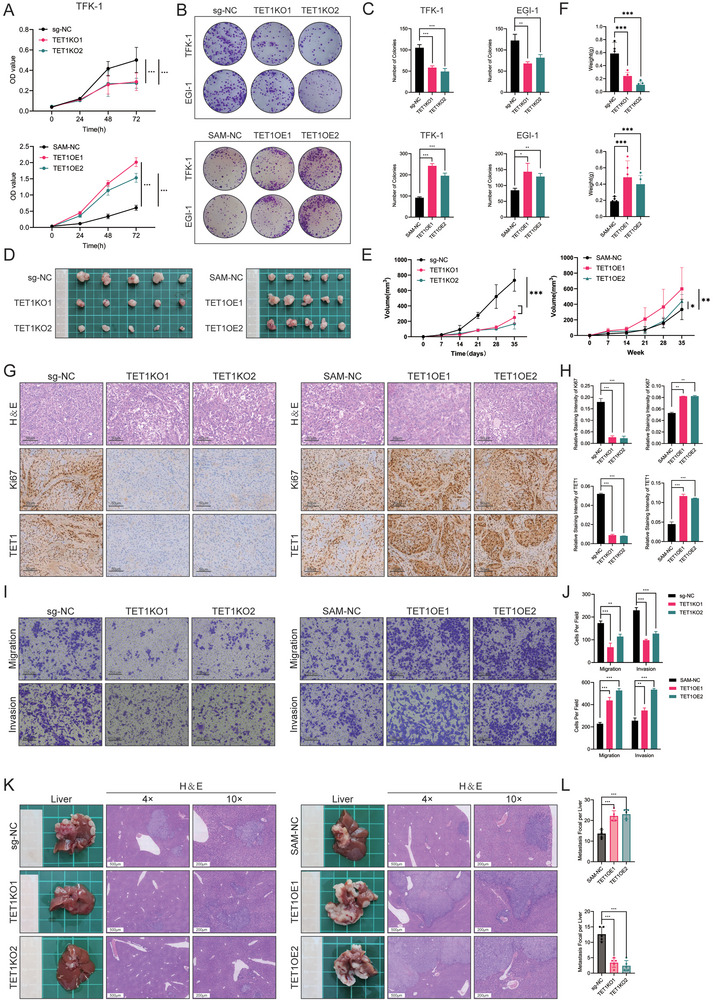
TET1 promotes cholangiocarcinoma proliferation, invasion, and migration in vivo and in vitro. A) CCK‐8 assays to examine the proliferation of TET1 knockout and overexpression of stably transfected TFK‐1 cell lines. B) Colony‐formation assays to examine the proliferation of TET1 knockout and overexpression of stably transfected TFK‐1 and EGI‐1 cell lines. C) Statistics on the number of clones in panel (B). D) Overview of tumors in transplanted xenografts with TET1KO or TET1OE and control cells (*n* = 5). E) Volume and F) weight of tumors in panel (D). G,H) H&E staining and Ki67 and TET1 G) staining and H) scoring of tumors in panel (D). I) Cell migration and invasion assay of TET1KO or TET1OE and control cells. J) Statistics of cells per field in panel (I). K) Overview and H&E staining of splenic injection of a CCA cell line model of liver metastasis. L) Statistics of metastasis focal per liver in panel (K) (*n* = 5). *P < 0.05, **p < 0.01, and ***p < 0.001.

Given the evidence that TET1 is highly expressed and promotes cholangiocarcinogenesis within human tissues and mouse CCA models, the effect of TET1 on the proliferation of stably transformed CCA cell lines was subsequently investigated. TET1 knockout (KO) and TET1 overexpression (OE) cell lines were initially constructed using clustered regularly interspaced short palindromic repeats (CRISPR)‐knockout and CRISPR‐active techniques in TFK‐1 and EGI‐1 cell lines. The cell counting kit‐8 (CCK‐8) assay demonstrated that TET1KO resulted in a deceleration of proliferation in TFK‐1 and EGI‐1 cells, whereas TET1OE led to an acceleration of proliferation (**Figure** **2**A; Figure S1A, Supporting Information). The results of the colony‐formation assays demonstrated a positive correlation between the number of clones and TET1 expression (Figure 2B,C). Xenograft tumor experiments in BALB/c‐nu mice demonstrated that TET1KO cell lines exhibited relatively slower tumor proliferation than controls (Figure 2D,E) and relatively more minor tumor weights (Figure 2F). Conversely, TET1OE cell lines displayed the opposite outcomes (Figure 2D–F). Furthermore, IHC staining demonstrated that the expression of Ki67 in TET1KO cell lines was lower than that in control xenograft tumors, whereas TET1OE resulted in increased expression of Ki67 (Figure 2G,H). These findings suggest that TET1 is pivotal in promoting CCA tumor growth and cell proliferation.

Subsequently, we conducted further assessments of the impact of TET1 on the invasive and metastatic potential of CCA in cell lines and mouse liver metastasis models. Wound healing assays demonstrated that TET1KO impeded the migratory capacity of CCA cells, whereas TET1OE facilitated the migration (Figure S1B,C, Supporting Information). Transwell cell migration and invasion assays demonstrated that TET1KO in TFK‐1 and EGI‐1 cell lines resulted in a reduction in cellular migratory and invasive abilities. Conversely, TET1OE in CCA cell lines enhanced invasive and migration capabilities (Figure 2I,J; Figure S2A,B, Supporting Information). Clinical studies have demonstrated that CCA displays a distinct propensity for metastasis to the liver.^[^ ^21^ ^]^ A liver metastasis model of spleen‐injected CCA cells was constructed to assess the effect of TET1 on liver metastasis in vivo. The results demonstrated that TET1KO reduced the number and area of liver nodules compared to the control group, whereas TET1OE led to an increase in the number of liver metastatic nodules (Figure 2K,L). In conclusion, TET1 plays a pivotal role in promoting CCA liver metastasis.

### 2.2 TET1 Is Dependent On Its Catalytic Activity to Promote CLDN3 Transcription in Cholangiocarcinoma

**Figure 3:**
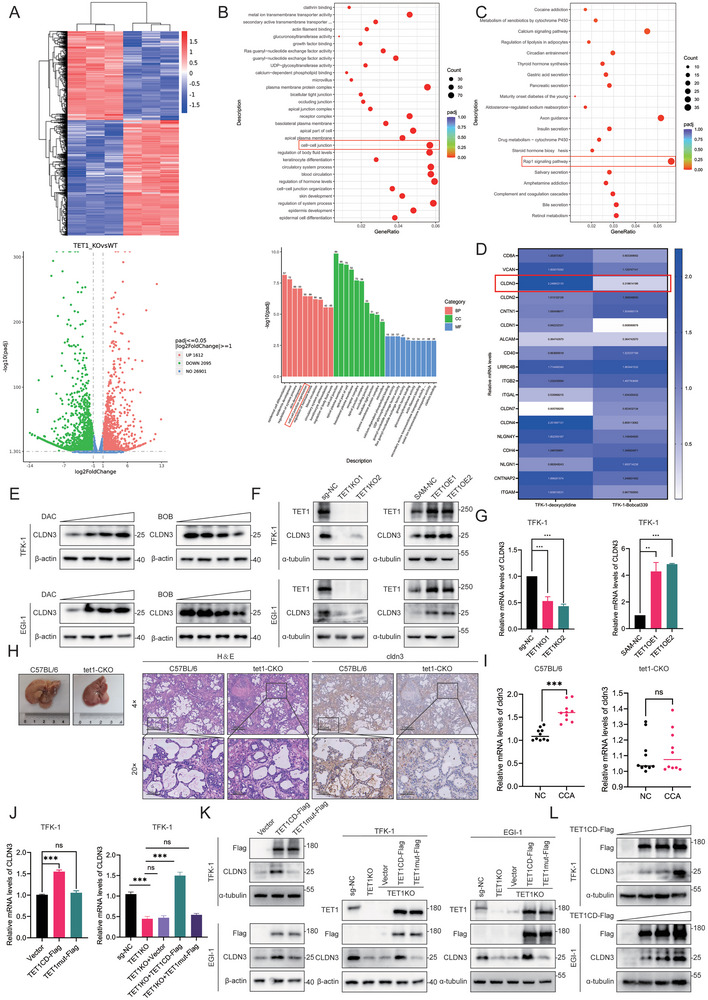
TET1 relies on its catalytic activity to promote CLDN3 transcription. A) Differential heat map (top) and volcano plot (bottom) of RNA‐Seq of TET1KO and control. B) Gene Ontology (GO) analysis of differential genes after TET1KO. C) Kyoto Encyclopedia of Genes and Genomes (KEGG) analysis of differential genes after TET1KO. D) qRT‐PCR assays to examine the gene expression level changes under the effect of DAC and BOB in the TFK‐1 cell line. E) WB assay for changes in CLDN3 protein levels with increasing concentrations of DAC and BOB. F) WB assays to detect changes in protein levels of CLDN3 expression in TET1KO or TET1OE and control cells. G) qRT‐PCR assays to detect changes in mRNA levels of CLDN3 expression in TET1KO or TET1OE and control cells. H) Overview of in situ CCA models in C57BL/6 and tet1‐CKO mice and IHC staining for H&E and cldn3. I) qRT‐PCR experiments were performed to detect cancer and paracancer CLDN3 mRNA levels in C57BL/6 and tet1‐CKO mice (*n* = 10). J) qRT‐PCR experiments to measure changes in mRNA levels of CLDN3 after steady transfection of TET1CD‐Flag and TET1mut‐Flag in the TFK‐1 cell line (left) and changes in mRNA levels of CLDN3 after reversion to TET1CD‐Flag and TET1mut‐Flag in the TET1KO cell line (right). K) WB experiments to measure changes in protein levels of CLDN3 after steady transfection of TET1CD‐Flag and TET1mut‐Flag in the TFK‐1 and EGI‐1 cell lines (left) and changes in mRNA levels of CLDN3 after reversion to TET1CD‐Flag and TET1mut‐Flag in the TET1KO cell line (right). L) WB assay to detect changes in CLDN3 protein levels with the amount of transfected TET1CD‐Flag plasmid. *P < 0.05, **p < 0.01, and ***p < 0.001; ns, not significant.

It has been demonstrated in the literature that TET1 catalyzes the demethylation of gene promoter regions, thereby activating downstream gene transcription.^[^ ^22^ ^]^ Consequently, we conducted transcriptome sequencing (RNA‐Seq) of the TET1KO CCA cell line TFK‐1 and its control cell lines. The results demonstrated that 1612 upregulated and 2095 downregulated genes were identified (**Figure** **3**A). Gene Ontology (GO) analysis indicated that the downregulated genes were predominantly enriched in cell–cell junctions, regulation of body fluid levels, circulatory system process, blood circulation, regulation of hormone levels, and membrane protein complexes (Figure 3B). The Kyoto Encyclopedia of Genes and Genomes (KEGG) analysis indicated that TET1KO predominantly influenced the Rap1 signaling pathway (Figure 3C). Following the synthesis of the results of the GO analysis and the KEGG analysis, TFK‐1 and EGI‐1 cells were incubated with Bobcat339 (BOB), a TET1 inhibitor, at a concentration of 33 µm, and decitabine (5‐Aza‐2′‐deoxycytidine, DAC), a methyltransferase inhibitor, at a concentration of 5 µm. The TFK‐1 and EGI‐1 cells were incubated for 48 h. Subsequently, quantitative real‐time polymerase chain reaction (qRT‐PCR) was performed to detect the expression of the top ten genes in each cluster obtained from the GO analysis and the expression of genes related to the Rap1 signaling pathway. The results demonstrated that the mRNA expression of CLDN3 was increased following DAC treatment and decreased following BOB treatment (Figure 3D; Figure S3A–E, Supporting Information). These findings suggest that TET1 may influence CLDN3 expression through its demethylation catalytic activity, affecting the EMT of CCA cells. To further test this hypothesis, we treated cells with DAC and BOB at increasing concentration gradients in TFK‐1 and EGI‐1 cell lines and performed WB experiments. The results demonstrated that CLDN3 expression increased with elevated DAC concentration and decreased with elevated BOB concentration (Figure 3E; Figure S3F, Supporting Information). Subsequently, we investigated whether CLDN3 expression was associated with TET1 expression in stable transformed CCA cell lines. The results demonstrated a positive correlation between TET1 expression with both protein and mRNA levels of CLDN3 (Figure 3F,G; Figure S3G, Supporting Information). These findings indicate that TET1 may positively regulate CLDN3 expression in CCA cell lines. We proceeded to investigate whether cldn3 expression remained regulated by tet1 in the mouse in situ CCA model. IHC and qRT‐PCR experiments demonstrated that cldn3 expression was diminished in the tet1‐CKO mouse model in the fourth week compared to that observed in the C57BL/6 mouse model (Figure 3H,I). The results indicate that TET1 regulates CLDN3 expression in vitro and in vivo.

To elucidate whether TET1 regulation of CLDN3 in vitro and in vivo is contingent on the demethylation catalytic activity of TET1, we constructed the overexpression plasmid phage‐TET1CD‐flag (TET1CD‐Flag), which encompasses the TET1 catalytic structural domain (TET1CD) solely, in addition to the TET1 enzyme inactivating mutant (H1652Y&D1654A) plasmids (TET1mut‐Flag). The TFK‐1 and EGI‐1 cell lines were stably transfected with TET1CD‐Flag and TET1mut‐Flag plasmids to examine the impact of TET1 regulation on CLDN3 expression. The results showed that the TET1CD‐Flag increased both the mRNA and protein expression of CLDN3, which was not observed in the TET1mut‐Flag‐transfected cells (Figure 3J,K (left); Figure S3H (left), Supporting Information). The transfection of TET1CD‐Flag plasmids in TET1KO cells reverted the mRNA and protein levels of CLDN3. In contrast, no such change was observed after the TET1mut‐Flag plasmids were transfected (Figure 3J,K (right); Figure S3H (right), Supporting Information). Transfection of the control plasmid (Vector), and 2, 4, and 6 µg of the TET1CD‐Flag plasmid in TFK‐1 and EGI‐1 cell lines, respectively, demonstrated that the expression of CLDN3 increased in proportion to the amount of transfected TET1CD‐Flag (Figure 3L). These findings indicate that TET1 plays a role in regulating CLDN3 expression in CCA, with the effect being dependent on its demethylation catalytic activity.

### 2.3 TET1 Facilitates Transcription by Targeting CLDN3 Promoter Demethylation

**Figure 4:**
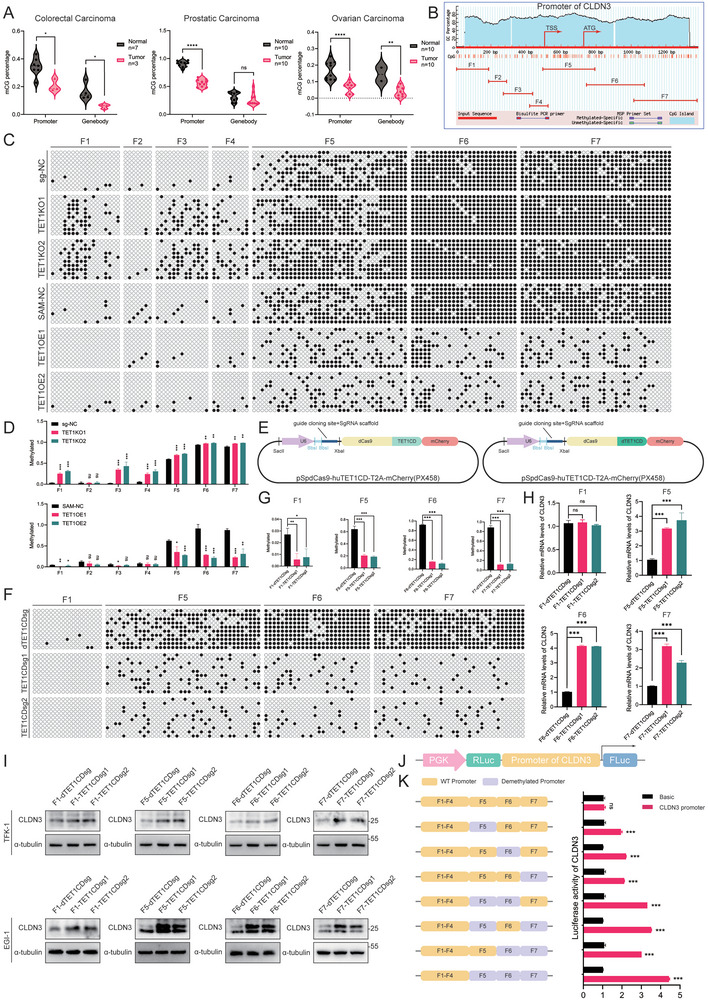
TET1 targets the CLDN3 promoter to promote transcription. A) https://ngdc.cncb.ac.cn/methbank/ website on methylation levels at different locations of the CLDN3 gene in different cancers. B) Schematic representation of the prediction and segmentation of the CLDN3 promoter by website https://urogene.org/methprimer2/. C,D) BSP assays to detect changes in methylation levels of different fragments of CLDN3 promoter in C) TET1KO and TET1OE cell lines and D) statistical plots (*n* = 10). E) Schematic representation of plasmids used for targeted demethylation. F,G) BSP assay to detect methylation changes in the CLDN3 promoter after F) targeting F1, F5, F6, and F7 demethylation and G) statistical plots (*n* = 10). H) Changes in mRNA levels of CLDN3 after targeting F1, F5, F6, and F7 demethylation detected by qRT‐PCR assays. I) Changes in protein levels of CLDN3 after targeting F1, F5, F6, and F7 demethylation detected by WB assays. J) Schematic diagram of the plasmid used for the dual luciferase assay. K) Dual luciferase assay to detect changes in fluorescence intensity with different degrees of methylation of the CLDN3 promoter. *P < 0.05, **p < 0.01, and ***p < 0.001; ns, not significant.

Prior experiments have demonstrated that TET1 plays a role in promoting CLDN3 transcription. Nevertheless, further investigation is required to ascertain whether these effects are achieved by targeting the CLDN3 promoter region for demethylation. We then utilized the https://ngdc.cncb.ac.cn/methbank/ website to analyze the variations in 5mC levels across distinct regions of the CLDN3 gene in various solid tumors. We found that the 5mC levels of CLDN3 were diminished in colon, prostate, and ovarian cancers in comparison to normal and paracancerous tissues. Additionally, these alterations were concentrated in the promoter region (**Figure** **4**A). The promoter region of CLDN3 was predicted using the promoter prediction website https://urogene.org/methprimer2/ and divided into seven segments to design BSP primers (Figure 4B). The methylation levels were detected in TET1KO and TET1OE cell lines in segments, and the results demonstrated differences in methylation level changes in F1, F5, F6, and F7 segments (Figure 4C,D). To further elucidate the relationship between alterations in methylation levels and expression within the CLDN3 promoter region, we employed the dCas9‐targeted DNA demethylation technique^[^ ^23^ ^]^ (Figure 4E) to develop single guide RNAs (sgRNAs) targeting the F1, F5, F6, and F7 segments, respectively, resulting in a reduction in DNA methylation levels (Figure 4F,G). Subsequently, alterations in the mRNA and protein levels of CLDN3 were observed following the targeting of DNA demethylation at the F1, F5, F6, and F7 segments, respectively. It was observed that the expression level of CLDN3 remained unaltered following the demethylation of the F1 segment. However, targeting the F5, F6, and F7 segments resulted in a notable elevation in CLDN3 expression (Figure 4H,I). To ascertain the function of the CLDN3 promoter, a dual luciferase reporter assay was conducted by inserting the CLDN3 promoter upstream of firefly luciferase (FLuc) and targeting distinct regions of the promoter for demethylation, respectively, and measuring the FLuc fluorescence intensity. The results demonstrated that promoter activity was enhanced following demethylation of F5, F6, and F7 (Figure 4J,K). The outcomes mentioned above indicate that TET1 demethylates the F5–F7 (from −16 to 512) region of the CLDN3 promoter, thereby directing it to facilitate CLDN3 transcription.

### 2.4 TET1 Utilizes Its Catalytic Activity to Promote EMT in Cholangiocarcinoma by Modulation of CLDN3 Expression

**Figure 5:**
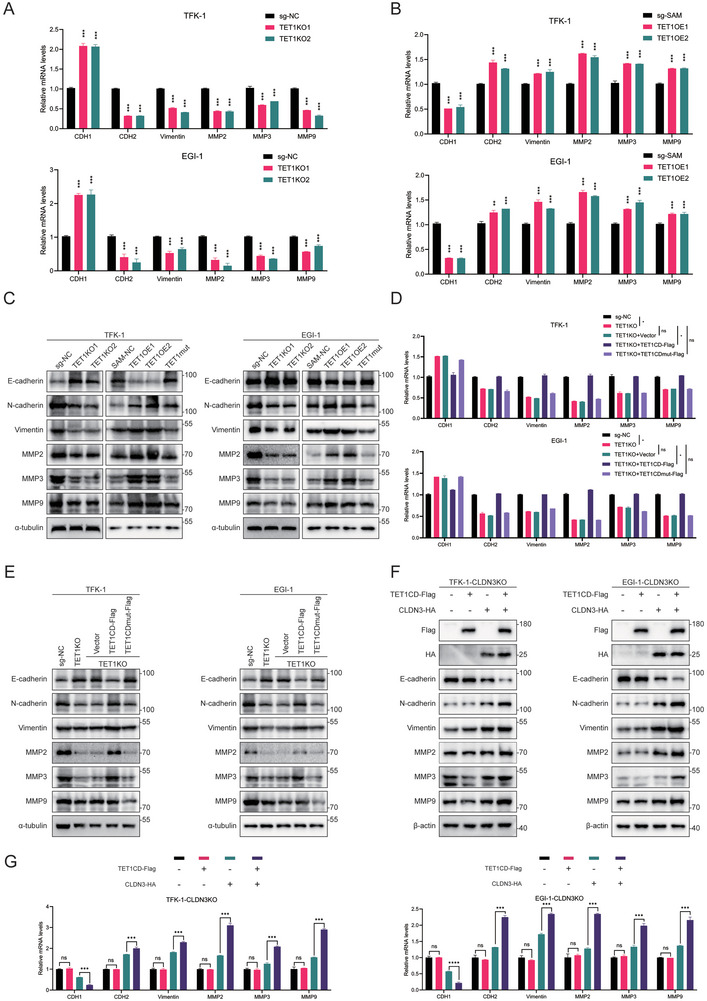
TET1‐dependent catalytic activity promotes EMT in cholangiocarcinoma. A) qRT‐PCR assays to detect changes in mRNA levels of EMT‐related indicators in TET1KO TFK‐1 and EGI‐1 cell lines. B) qRT‐PCR assays to detect changes in mRNA levels of EMT‐related indicators in TET1OE TFK‐1 and EGI‐1 cell lines. C) WB assays to detect changes in protein levels of EMT‐related indicators in TET1KO as well as TET1OE/mutated TFK‐1 and EGI‐1 cell lines. D) qRT‐PCR assays were performed to detect changes in the levels of EMT‐related mRNAs in TET1KO cell lines, restoring TET1CD and TET1mut plasmids in TFK‐1 and EGI‐1 cell lines. E) WB assays were performed to detect changes in the levels of EMT‐related proteins in TET1KO cell lines, restoring TET1CD and TET1mut plasmids in TFK‐1 and EGI‐1 cell lines. F) WB assays were performed to detect changes in the levels of EMT‐related proteins after overexpression of TET1CD in CLDN3KO cell lines and after rescuing to CLDN3. G) qRT‐PCR assays were performed to detect changes in the levels of EMT‐related mRNAs after overexpression of TET1CD in CLDN3KO cell lines and after rescuing to CLDN3. *P < 0.05, **p < 0.01, and ***p < 0.001; ns, not significant.

Changes in the expression of EMT‐related indicators were examined in the TET1KO and TET1OE stably transient cell lines. The results showed that E‐cadherin mRNA expression was increased, and N‐cadherin, vimentin, MMP2, MMP7, and MMP9 mRNA expressions were decreased after TET1KO, implying a weakening of EMT (**Figure** **5**A). In contrast, overexpression of TET1 resulted in decreased mRNA expression of E‐cadherin, increased mRNA expression of N‐cadherin, vimentin, MMP2, MMP7, and MMP9, and enhanced EMT (Figure 5B). WB experiments on the expression levels of EMT‐related proteins resulted in comparable outcomes and revealed that TET1 mutants do not lead to changes in the expression of EMT indicators (Figure 5C). The TET1CD‐Flag and TET1mut‐Flag plasmids were reverted in TET1KO cell lines and then examined for changes in mRNA and protein levels of EMT‐related indicators. The results demonstrated that, compared to the TET1KO cell line, the restoration of TET1CD expression resulted in a suppression of E‐cadherin expression. In contrast, N‐cadherin, vimentin, MMP2, MMP7, and MMP9 expressions were restored. No changes were observed in the transfected TET1mut‐Flag plasmid (Figure 5D,E). The results mentioned above indicate that TET1 exerts its role in promoting EMT in CCA through its demethylation catalytic activity.

Having been previously demonstrated that TET1 promotes CLDN3 transcription by targeting CLDN3 promoter demethylation, we investigated whether the EMT‐promoting function of TET1 in CCA is realized through CLDN3. The role of CLDN3 in the proliferation, invasion, and metastasis of CCA was initially validated. CLDN3OE and CLDN3KO cell lines were constructed to investigate the impact of CLDN3 on EMT‐related indicators. The results demonstrated that in CLDN3KO TFK‐1 and EGI‐1 cell lines, the mRNA expression of E‐cadherin was increased. In contrast, the mRNA expressions of N‐cadherin, vimentin, MMP2, MMP7, and MMP9 decreased, leading to an attenuation of EMT (Figure S4A, Supporting Information). Conversely, when CLDN3 was overexpressed, there was a notable decline in the mRNA expression of E‐cadherin, accompanied by a surge in the mRNA expressions of N‐cadherin, vimentin, MMP2, MMP7, and MMP9, which collectively indicated a propensity for enhanced EMT (Figure S4B, Supporting Information). WB experiments to detect the expression levels of EMT‐related proteins in TFK‐1 and EGI‐1 cell lines yielded identical results to those observed at the mRNA level (Figure S4C,D, Supporting Information), thereby demonstrating that CLDN3 itself is a crucial promoter of EMT in CCA cells. Xenograft tumor experiments in BALB/c‐nu mice demonstrated that CLDN3KO cell lines exhibited relatively slower tumor proliferation compared to the control small‐guide RNA (sg‐NC) group (Figure S4E,F, Supporting Information) and lighter tumor weight after the experiments (Figure S4G, Supporting Information). Conversely, CLDN3OE xenograft tumors demonstrated accelerated growth (Figure S4E,F, Supporting Information). Furthermore, IHC staining revealed that the expressions of Ki67 in CLDN3KO cell lines were lower than that in control xenograft tumors, whereas CLDN3OE promoted the expression of Ki67 (Figure S4H, Supporting Information). Transwell cell migration and invasion assays demonstrated that the CLDN3KO TFK‐1 cell line diminished cell migration and invasiveness. In contrast, CLDN3OE made CCA cells exhibit more vital invasion and migration ability. The above results suggest that CLDN3 may promote CCA tumor growth, invasion, and migration. Control and CLDN3‐HA vectors were transfected in CLDN3KO cell line, and then TET1CD‐Flag and its control plasmid were overexpressed separately, and the expressions of EMT‐related proteins and mRNAs were detected. The results demonstrated that TET1CD overexpression was unable to enhance CCA EMT following CLDN3KO. Conversely, the effect of TET1CD overexpression on enhancing CCA EMT was restored following reversion to CLDN3 (Figure 5F,G). The results above indicated that CLDN3 could promote the proliferation, invasion, and migration of CCA cells and play a pivotal role in promoting CCA EMT by TET1.

### 2.5 PPM1G Destabilizes the TET1 Protein by Catalyzing TET1 Dephosphorylation

**Figure 6:**
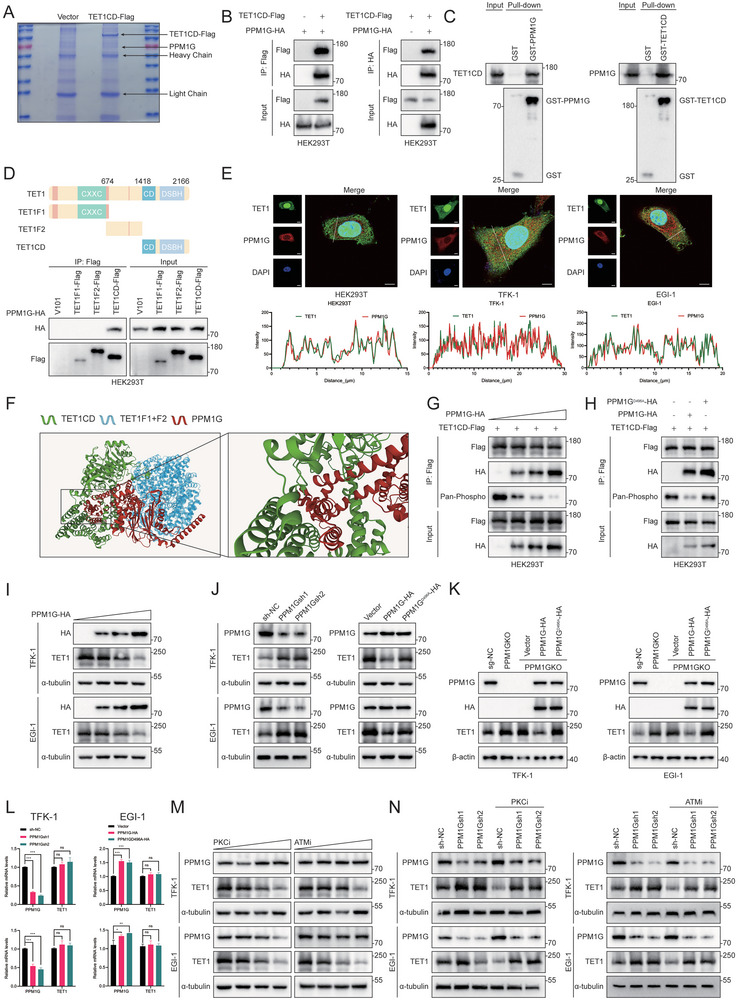
PPM1G destabilizes TET1 protein by catalyzing TET1 dephosphorylation. A) Caumas brilliant blue staining of gels used for HPLC‐MS/MS experiments. B) HEK293T cells were transfected with the corresponding plasmids and then immunoprecipitated with anti‐HA or anti‐Flag antibodies and immunoblotted with anti‐Flag or anti‐HA antibodies, respectively. C) Cell lysates from HEK293T cells were co‐incubated with beads with GST, GST‐TET1CD, or GST‐PPM1G. Immunoblotting was performed to analyze pull‐down samples and whole cell lysates. D) Schematic representation of TET1 segmentation and immunoprecipitation analysis of different segments bound to PPM1G. E) TET1 (green) and PPM1G (red) were visualized by confocal microscopy in HEK293T, TFK‐1, and EGI‐1 cells transfected with Flag‐TET1CD and HA‐PPM1G plasmids and immunofluorescence was performed. Line intensity maps show co‐localization between TET1 and PPM1G. Images represent at least *n* = 5 imaged cells. Scale bar, 5 µm. F) Computer‐performed molecular docking simulation of PPM1G with TET1. In the figure, the green protein represents the TET1 catalytic structural domain, the blue protein represents the other regions of TET1 except the catalytic structural domain, and the red protein represents the PPM1G protein (http://autodock.scripps.edu/). G) HEK293T cells were transfected with the same mass of TET1CD‐Flag plasmid and with different masses of PPM1G‐HA plasmid, and TET1CD was pulled down with Flag antibody and the phosphorylation was detected. H) HEK293T cells were transfected with TET1CD‐Flag plasmid and with PPM1G‐HA as well as PPM1GD496A‐HA plasmids, and TET1CD was pulled down with Flag antibody, and the degree of phosphorylation was detected. I) WB assays were used to detect the expression of TET1 after the transfection of TFK‐1 and EGI‐1 cells with plasmids encoding PPM1G‐HA at different qualities. J) TET1 expression was detected by WB assay in PPM1G knockout and overexpressed/mutated TFK‐1 and EGI‐1 stably transfected cell lines. K) WB assays were employed to detect TET1 expression following the restoration of PPM1G and mutant PPM1G in PPM1GKO TFK‐1 and EGI‐1 cell lines. L) qRT‐PCR assays detected TET1 mRNA expression in PPM1G knockout and overexpression/mutant TFK‐1 and EGI‐1 cell lines. M) TFK‐1 and EGI‐1 cell lines were treated with gradually increasing concentrations of PKCi and ATMi. WB detected and TET1 expression. N) PPM1G knockout TFK‐1 and EGI‐1 cell lines were treated with gradually increasing concentrations of PKCi and ATMi. WB detected the TET1 expression. *P < 0.05, **p < 0.01, and ***p < 0.001; ns, not significant.

As previously stated, TET1 is highly expressed in CCA and correlates with the prognosis. It has a strong promotion effect on the proliferation, invasion, and migration of CCA cells. Therefore, inhibiting CCA progression by inhibiting the expression of TET1 or promoting the degradation of TET1 in CCA may become a new therapeutic modality. The literature has previously documented that TET1 exhibits enhanced stability and resistance to degradation following phosphorylation.^[^ ^17^ ^]^ Consequently, catalytic dephosphorylation of TET1 may represent a novel approach to facilitating its degradation. Due to the molecular weight of TET1 being too large to accommodate the TET1 full‐length overexpression plasmid for immunoprecipitation (IP), we employed HPLC‐MS/MS in the TFK‐1 cell line stably transfected with the TET1CD‐Flag to identify proteins that might bind to TET1. The results demonstrated that serine dephosphorylase PPM1G was immunoprecipitated by TET1 (**Figure** **6**A). The PPM1G‐hemagglutinin (HA) plasmid was constructed and co‐transfected with TET1CD‐Flag in HEK293T cells. Exogenous Co‐immunoprecipitation (Co‐IP) experiments were performed using labeled antibody HA or Flag. The results demonstrated an interaction between TET1 and PPM1G (Figure 6B). To further elucidate the nature of the interaction between TET1 and PPM1G, we constructed Glutathione S‐Transferases (GST)‐TET1CD and GST‐PPM1G plasmids using the pGEX‐4T‐1 vector. These plasmids were then transfected into *Escherichia coli* BL21, which induced the expression of TET1 and PPM1G proteins, respectively. The GST fusion proteins were purified and subjected to a GST pull‐down assay. The results demonstrated the direct interaction between TET1 protein and PPM1G (Figure 6C). To ascertain whether PPM1G binds to regions other than the TET1CD structural domain, we divided TET1 into three distinct segments: the CXXC region (TET1F1), the unknown functional region other than CD and CXXC (TET1F2), and the CD region (TET1CD), based on its independent functional characteristics. We then constructed overexpression plasmids for each of these segments. HEK293T cells were transfected with plasmids encoding PPM1G‐HA and TET1 segments, and Co‐IP experiments were conducted. The results demonstrated that no additional structural domains were involved in the interaction between PPM1G and TET1 (Figure 6D). To further elucidate the cellular localization of TET1 and PPM1G, HEK293T cells and CCA cell lines TFK‐1 and EGI‐1 were transfected with TET1CD‐Flag and PPM1G‐HA plasmids, respectively. The results demonstrated that PPM1G was predominantly localized in the cytoplasm, with minimal expression in the nucleus, whereas TET1 exhibited a more distributed pattern, with a predominant presence in the nucleus. TET1 was distributed in the cytoplasm and nucleus, with the majority present in the nucleus. However, Merge image demonstrated that TET1 had a clear co‐localization with PPM1G in the cytoplasm (Figure 6E). Molecular docking was employed to simulate the binding of PPM1G to the TET1 protein. The results demonstrated that PPM1G binds to the catalytic domain of TET1 (Figure 6F). After establishing the mutual binding relationship between TET1 and PPM1G, we proceeded to investigate whether PPM1G was capable of catalyzing TET1 dephosphorylation and whether it influenced TET1 expression. HEK293T cells were transfected with a control plasmid, a PPM1G‐HA plasmid (2, 4, and 6 µg), and a TET1CD‐Flag plasmid, respectively. Co‐IP then detected the degree of phosphorylation of TET1. The results demonstrated that the degree of TET1 phosphorylation decreased with the increase of PPM1G expression (Figure 6G). The D496A mutant of PPM1G is unable to undergo phosphorylation.^[^ ^24^ ^]^ The PPM1GD496A‐HA plasmid was constructed, and Co‐IP was performed after co‐transfection with TET1CD‐Flag in HEK293T cells. The results showed that the PPM1G mutant did not affect the TET1 phosphorylation degree (Figure 6H). To clarify whether PPM1G can endogenously affect TET1 in CCA cell lines, TFK‐1 and EGI‐1 were transfected with a control plasmid, 2, 4, and 6 µg of PPM1G‐HA plasmid, respectively, and changes in endogenous TET1 expression were subsequently detected. The results demonstrated a gradual decrease in the expression of endogenous TET1 with an increase in PPM1G expression (Figure 6I). Stably transformed CCA cell lines were constructed with short hairpin RNA (shRNA) and PPM1G‐HA and PPM1GD496A‐HA plasmids to create knockdown (PPM1Gsh1, PPM1Gsh2), overexpression, and mutant cell lines, respectively. The expression of TET1 was detected in the stably transfected cell lines. WB results demonstrated that the expression of TET1 was increased in the PPM1G knockdown cell lines. In contrast, the expression of TET1 was decreased in the overexpressed cell lines, and there was no discernible trend in the change of TET1 expression in the cell lines with mutated PPM1G catalytic activity (Figure 6J). PPM1GKO cell lines were constructed and reverted to PPM1G‐HA and PPM1GD496A‐HA plasmids to ascertain the impact of these alterations on TET1 expression. The results demonstrated that TET1 expression increased following PPM1GKO but returned to control levels upon reverting to PPM1G, whereas the PPM1G mutant exhibited no such effect (Figure 6K). To eliminate the possibility that PPM1G regulates TET1 at the transcriptional level, we examined the mRNA levels of TET1 in PPM1G knockdown and overexpressed stable‐transformed cell lines. The results demonstrated that the mRNA expression of TET1 remained unaltered in response to alterations in PPM1G levels (Figure 6L). This result rules out the possibility that PPM1G affects TET1 expression by regulating transcription. A review of the literature revealed that the phosphatases that catalyze TET1 phosphorylation are mainly classified into the protein kinase C (PKC) family and the serine–threonine protein kinase ataxia–telangiectasia‐mutated protein (ATM) family.^[^ ^17^ ^]^ Accordingly, staurosporine (PKCi), which belongs to the PKC‐inhibiting family, and AZD0156 (ATMi), which inhibits the ATM family, were selected for testing to determine whether the use of small‐molecule inhibitors could alter the protein level of TET1. PKCi at a concentration of 6 nm and ATMi at 2 µm were co‐incubated with TFK‐1 and EGI‐1 cells for 8 h. WB results demonstrated that TET1 protein levels decreased with increasing concentrations of PKCi and ATMi (Figure 6M). The co‐incubation of PPM1G knockdown stable‐transformed cell lines with PKCi and ATMi demonstrated that, in accordance with the reduction in TET1 expression level resulting from PPM1G, the introduction of the inhibitor led to a further decline in TET1 expression (Figure 6N). The aforementioned results indicate that the binding of PPM1G to the TET1 protein enhances its instability and accelerates its degradation, thereby reducing the level of TET1 protein in CCA.

### 2.6 PPM1G Elevates 5mC Levels at the CLDN3 Promoter and Suppresses CLDN3 Transcription via the Inhibition of TET1 Stability

**Figure 7:**
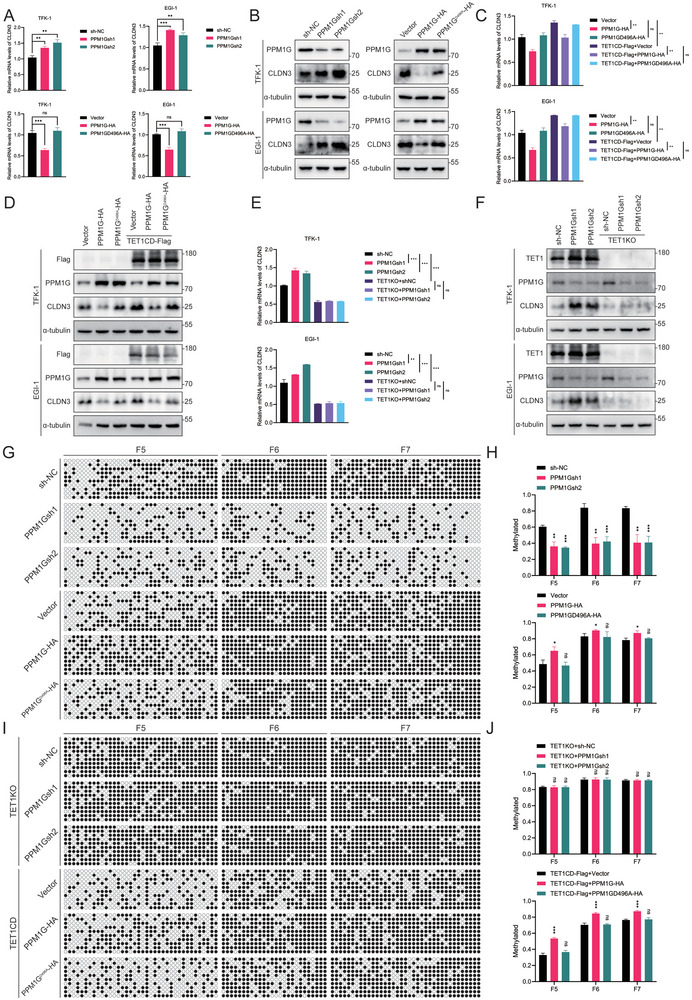
PPM1G suppresses CLDN3 transcription by depending on TET1. A) qRT‐PCR assays detect mRNA levels of CLDN3 in PPM1G knockdown and overexpressed/mutated TFK‐1 and EGI‐1 cell lines. B) WB assays were performed to detect protein levels of CLDN3 in PPM1G knockdown and overexpressed/mutated TFK‐1 and EGI‐1 cell lines. C) Transfection of TET1CD in PPM1G overexpressing/mutated TFK‐1 and EGI‐1 cell lines and detection of CLDN3 mRNA levels by qRT‐PCR. D) Transfection of TET1CD in PPM1G overexpressing/mutated TFK‐1 and EGI‐1 cell lines and detection of CLDN3 protein levels by WB. E) The knockout of TET1 in PPM1G knockdown TFK‐1 and EGI‐1 cell lines and detection of CLDN3 mRNA levels by qRT‐PCR. F) The knockout of TET1 in PPM1G knockdown TFK‐1 and EGI‐1 cell lines and detection of CLDN3 protein levels by WB. G,H) BSP assay to detect methylation level of F5–F7 fragment of CLDN3 promoter in PPM1G knockdown, overexpression/mutant cell lines, and statistical analysis. I,J) BSP assay to detect the methylation level of the F5–F7 region of the CLDN3 promoter region in PPM1G knockdown and knockout of TET1 and PPM1G overexpression/mutation and transfection of TET1CD and its statistical analysis. *P < 0.05, **p < 0.01, and ***p < 0.001; ns, not significant.

Given that it was evident that PPM1G binds to and destabilizes the TET1 protein, we proceeded to investigate whether this would result in alterations to downstream CLDN3 expression. CLDN3 mRNA and protein levels were examined in PPM1G knockdown, overexpression, and mutated stably transient cell lines. The results demonstrated that CLDN3 expression levels were elevated following PPM1G knockdown and reduced following PPM1G overexpression. However, the change in CLDN3 levels following PPM1G mutation was not statistically significant (**Figure** **7**A,B). To ascertain that the observed changes in CLDN3 levels were due to the regulation of TET1 by PPM1G rather than the direct regulation by PPM1G, we stably transfected the TET1CD‐Flag based on PPM1G overexpression as well as the mutated stably transfected cell lines in order to revert to the expression of TET1. The results demonstrated that the mRNA and the protein level of CLDN3 reverted with the increase in the expression of TET1 (Figure 7C,D). The knockout of TET1 in PPM1G knockdown stable‐transformed cell lines revealed a significant decrease in CLDN3 expression. However, this difference lost statistical significance compared to the control group (Figure 7E,F). The results indicated that the alterations in CLDN3 levels resulting from modifications in PPM1G levels were contingent on the intermediate molecule TET1.

Subsequently, an investigation was conducted to ascertain whether the inhibitory effect of PPM1G on TET1 protein levels could increase methylation levels within the downstream CLDN3 promoter region. The methylation level of the CLDN3 promoter F5–F7 (from −16 to 512) segments was examined by BSP in PPM1G‐stabilized cell lines. The results demonstrated that the methylation level of the CLDN3 promoter region exhibited a negative correlation with PPM1G levels, decreasing as PPM1G levels decreased and increasing as PPM1G levels increased. Additionally, the change in methylation degree caused by the PPM1G mutant was not statistically significant (Figure 7G,H). To ascertain that PPM1G is unable to induce alterations in CLDN3 promoter levels independently but rather depends on the demethylation of TET1, we examined CLDN3 promoter methylation levels in PPM1G knockdown with TET1 knockout as well as PPM1G overexpression and mutant with overexpression of TET1CD in stably transduced cell lines. Upon overexpression of TET1CD, CLDN3 promoter methylation levels were decreased (Figure 7I,J). The aforementioned results indicated that the inhibitory effect of PPM1G on TET1 could further elevate the downstream CLDN3 promoter methylation level and inhibit CLDN3 transcription.

### 2.7 A Combination of Phosphatase Inhibitors and TET1 Inhibitors Can Inhibit Cholangiocarcinogenesis

**Figure 8:**
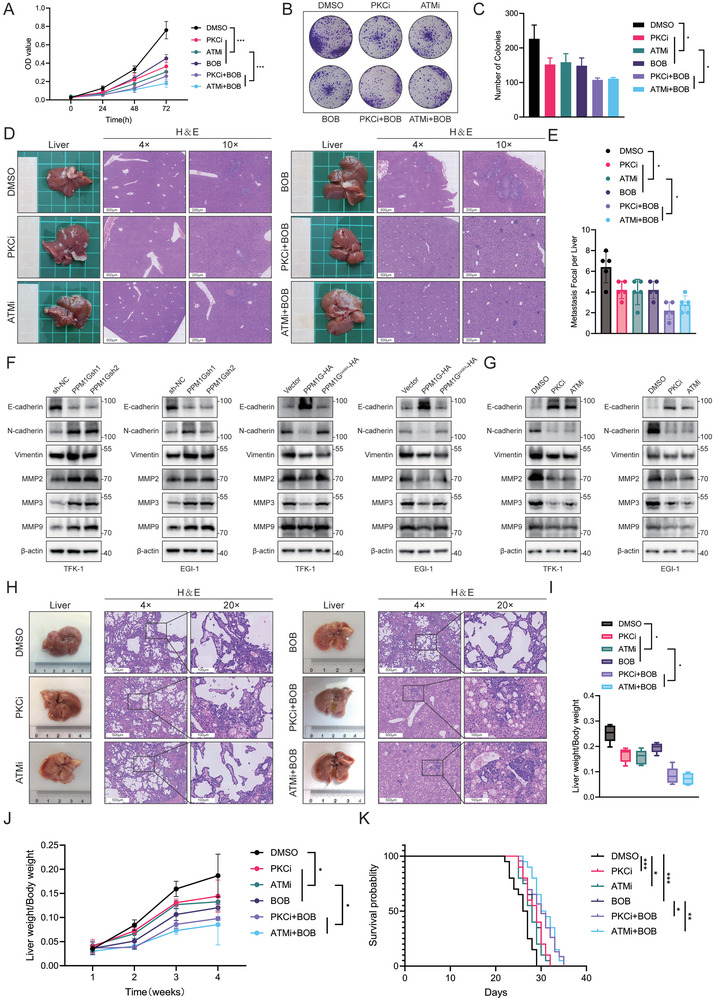
Combination of a phosphatase inhibitor and a TET1 inhibitor suppresses EMT in CCA. A) CCK‐8 assay was performed to detect the proliferation of TFK‐1 cells under the effect of different drugs (DMSO, PKCi, ATMi, BOB, PKCi+BOB, and ATMi+BOB). B,C) A colony‐formation assay was performed to detect the proliferation of TFK‐1 cells under the effect of different drugs (DMSO, PKCi, ATMi, BOB, PKCi+BOB, and ATMi+BOB) and its statistics. D) Overview and H&E staining of splenic injections in a liver metastatic CCA model under the effect of different drugs. E) Statistics of metastasis focal per liver in panel (D) (*n* = 5). F) WB assays were performed to detect the expression of EMT‐related proteins in PPM1G knockdown and overexpressed/mutated TFK‐1 and EGI‐1 cell lines. G) WB assay was performed to detect the expression of EMT‐related proteins in TFK‐1 and EGI‐1 cell lines under the effect of different drugs. H) Overview and H&E staining of in situ CCA models in mice under the effect of different drugs at 4 weeks. I) Liver weight‐to‐body weight ratio in a mouse model of in situ CCA under the effect of different drugs (*n* = 5). J) Liver weight‐to‐body weight ratio growth curves in a mouse model of in situ CCA under the effect of different drugs. K) Survival curves in a mouse model of in situ CCA under different drugs (*n* = 20). *P < 0.05, **p < 0.01, and ***p < 0.001; ns, not significant.

The experiments described in the previous section have elucidated that PPM1G can catalyze TET1 dephosphorylation and destabilize TET1, thereby weakening its targeting demethylation of the CLDN3 promoter region. Consequently, we investigated whether it was possible to delay CCA development by combining a phosphatase inhibitor and a TET1 inhibitor. The drugs were divided into five groups: a dimethyl sulfoxide (DMSO) control, PKCi, ATMi, BOB, PKCi+BOB, and ATMi+BOB. The proliferation of CCA cells was quantified using the CCK‐8 assay following the co‐incubation of several groups of drugs with TFK‐1 and EGI‐1. The results demonstrated that PKCi, ATMi, and BOB could inhibit the proliferation of cholangiocarcinoma cells. However, the inhibition of CCA proliferation was more pronounced when PKCi, ATMi, and BOB were combined (**Figure** **8**A; Figure S5A, Supporting Information). Additionally, colony‐formation assays demonstrated that combining PKCi, ATMi, and BOB reduced the number of clones formed (Figure 8B,C; Figure S5B,C, Supporting Information). In order to investigate the effect of drug action on cholangiocarcinoma cell invasion in vivo, the impact of the drugs on liver metastasis in vivo was assessed after intraperitoneal injection of several groups of medications into the constructed spleen‐injected CCA cell liver metastasis BALB/c‐nu mouse model, respectively. The results demonstrated that PKCi, ATMi, and BOB injections reduced the number and area of liver nodules compared to the control group. Conversely, the PKCi+BOB and ATMi+BOB groups exhibited a reduction in the number of liver metastatic nodules (Figure 8D,E). This study aimed to detect the changes in EMT‐related indexes in PPM1G stable‐transformed cholangiocarcinoma cell lines. The results demonstrated that following PPM1G knockdown, E‐cadherin levels were decreased, while N‐cadherin, vimentin, MMP2, MMP7, and MMP9 levels were increased, indicating that EMT was enhanced. Conversely, the trend of the indicators following PPM1G overexpression was reversed, with EMT being weakened and no such change observed in the PPM1G mutant (Figure 8F; Figure S5D, Supporting Information). Using PKCi and ATMi in cell treatment showed a discernible trend of EMT weakening (Figure 8G; Figure S5E, Supporting Information). In conclusion, the phosphatase inhibitor has been demonstrated to play a pivotal role in the inhibition of EMT in both in vivo and in vitro models. Furthermore, adding a TET1 inhibitor has enhanced the inhibitory effect.

Subsequently, we investigated the impact of phosphatase inhibitors and TET1 inhibitors on the progression of the in situ cholangiocarcinoma model. The in situ CCA model was constructed in C57BL/6 mice, and several groups of drugs were injected intraperitoneally into the mice. Five mice were executed at weeks 2–4, and H&E staining was performed to observe the growth of CCA under different drug treatments. The remaining rats were marked for the time of death (*n* = 20) to facilitate the observation of the effects of the drug treatments on the mice over time. These results demonstrated that CCA in the PKCi‐, ATMi‐, and BOB‐injected mice exhibited a slower malignancy progression than in the control group. Furthermore, the progression of CCA in the PKCi+BOB and ATMi+BOB groups was even slower (Figure 8H; Figure S6A,B, Supporting Information). The statistical analysis of liver weight relative to body weight in mice revealed that the liver weight/body weight ratios were significantly lower in mice that received PKCi, ATMi, or BOB injections, with the weakest ratios observed in mice that received both PKCi and BOB (PKCi+BOB) or ATMi and BOB (ATMi+BOB) injections (Figure 8I,J). A comparison of the survival curves of mice in the various experimental groups revealed that those injected with PKCi, ATMi, and BOB survived for a more extended period, with the mice in the PKCi+BOB and ATMi+BOB groups exhibiting the most extended survival. In conclusion, the phosphatase inhibitors PKCi and ATMi and the TET1 inhibitor BOB can impede the progression of CCA. However, the combination of PKCi or ATMi with BOB has been observed to extend the survival of mice, thereby improving the overall prognosis.

**Figure 9:**
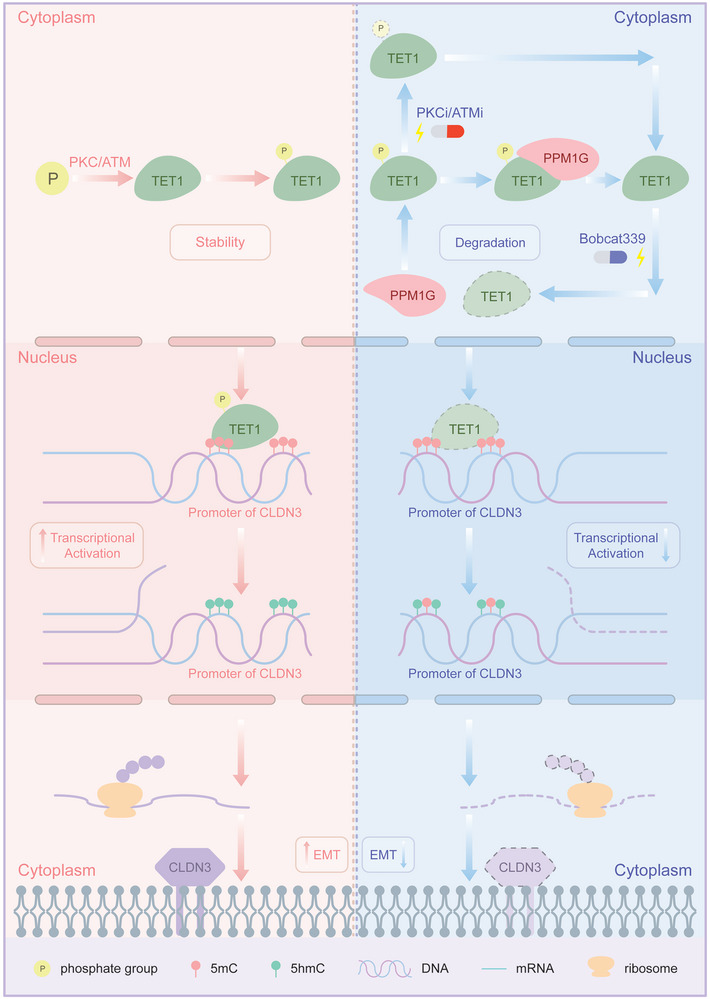
Model diagram of PPM1G–TET1–CLDN3.

The results of our experiments demonstrated that TET1 plays a role in promoting EMT in CCA cells by activating CLDN3 transcription by targeting the CLDN3 promoter region from −16 to 512 for demethylation. Furthermore, our findings revealed that PPM1G, in addition to PKCi and ATMi, can inhibit the proto‐oncogenic effect of TET1 by decreasing the stability of the TET1 protein. The complete mechanism diagram is presented in **Figure** **9**.

## 3 Discussion

Epigenetic modification is intimately associated with tumor development.^[^ ^25^ ^]^ It primarily regulates gene function and expression level through DNA methylation, histone modification, noncoding RNA regulation, and chromatin structure remodeling, thereby influencing tumor progression.^[^ ^26^ ^]^ Drugs targeting epigenetics have been gradually applied to treat malignant tumors.^[^ ^27^ ^]^ Commonly used drugs include DNA methyltransferase inhibitors^[^ ^28^ ^]^ and histone deacetylase inhibitors.^[^ ^29^ ^]^ However, these drugs still have many shortcomings, and further research is needed for widespread clinical application.^[^ ^30^ ^]^

TET1 protein is a crucial epigenetic regulatory enzyme within the TET protein family of human α‐ketoglutarate (α‐KG) oxidases. It can facilitate DNA demethylation through two distinct pathways: active and passive demethylation,^[^ ^31^ ^]^ thereby completing the reversal of DNA methylation and activating gene expression.^[^ ^32^ ^]^ TET1 exhibits distinct genetic properties in different tumor types. Its role as a proto‐oncogene or an oncogene is contingent upon the downstream genes and signaling pathways regulated in that tumor.^[^ ^33^ ^]^ For example, TET1 is a proto‐oncogene in T‐cell acute lymphoblastic leukemia (T‐ALL),^[^ ^34^ ^]^ whereas it exhibits oncogene properties in acute myeloid leukemia (AML) and correlates with patients’ prognosis.^[^ ^11^ ^]^ In breast cancer, TET1 exhibited a notable decrease in expression levels in tumors of estrogen‐pregnant hormone receptor‐positive patients. In contrast, it demonstrated a marked increase in expression levels in tumors of triple‐negative breast cancer patients.^[^ ^35^ ^]^ It has been proposed that TET1 can be employed as a specific target in the clinical treatment of tumors. For instance, olaparib, a clinically approved poly ADP ribose polymerase (PARP) inhibitor, has been demonstrated to induce 5hmC labeling loss, thereby inhibiting the proliferation of T‐ALL leukemia cells.^[^ ^36^ ^]^ The role of TET1 in CCA has been the subject of several studies. For example, a comparative study conducted in 2017 revealed that TET1 expression in CCA is inhibited by miR‐191, which maintains the CpG‐rich methylated region of the transcription start site of the p53 gene methylated, resulting in reduced p53 expression and diminished anticancer activity of p53.^[^ ^37a^ ^]^ A 2021 study investigated the effects of α‐KG and dimethyl‐α‐KG (DM‐α‐KG) on CCA cells. These co‐substrates of the TET1 dioxygenase were found to promote the formation of 5hmC and malignant tumorigenesis in CCA cells.^[^ ^37b^ ^]^ While numerous studies have examined the function of TET1 in CCA, the potential of leveraging TET1's epigenetic capabilities to impede cholangiocarcinoma progression remains uncharted territory.

Based on the abovementioned research, we initially ascertained that TET1 was markedly expressed in cholangiocarcinoma tissues and correlated with prognosis through a tissue microarray and an in situ cholangiocarcinoma mouse model. The in situ cholangiocarcinoma model mice were constructed in C57BL/6 mice and tet1‐CKO mice, which demonstrated prolonged survival and an improved prognosis in TET1 knockout mice. Subsequently, in vivo and in vitro experiments showed that TET1 promotes cholangiocarcinoma proliferation, invasion, and metastasis. Given that TET1 is primarily responsible for the demethylation of downstream gene promoter regions, which subsequently activates gene expression, we performed RNA‐Seq experiments to ascertain the impact of TET1KO on downstream gene expression. The data analysis indicated that most genes exhibiting downregulation were enriched in cell–cell junctions and membrane protein complexes. DNA methyltransferase and TET1 inhibitors were employed to screen for the TET1‐dependent regulation of the catalytic activity of the target gene CLDN3. The positive regulation of CLDN3 by TET1 was verified in TET1KO and TET1OE cell lines. Furthermore, it was highly correlated with the effects of the methylation inhibitor DAC and the TET1 inhibitor BOB. The expression of cldn3 was examined in an in situ CCA model constructed in C57BL/6 mice and tet1‐CKO mice. It was found that tet1 also regulated the expression of cldn3. To further elucidate the relationship between TET1‐mediated CLDN3 regulation and its demethylation activity, we constructed an overexpression plasmid, TET1CD‐Flag, which contains only the catalytic domain of TET1. In addition, we constructed the TET1 enzyme inactivation mutation (H1652Y&D1654A) plasmid (TET1mut‐Flag) and transfected CCA cell lines with TET1CD‐Flag plasmid, respectively. The results showed that CLDN3 expression increased with the amount of transfected TET1CD‐Flag plasmid, and TET1CD restored CLDN3 expression that TET1KO reduced. In contrast, there was no statistically significant change in CLDN3 expression levels in cells transfected with TET1mut‐Flag.

In conclusion, our experiments demonstrate that TET1 exerts its regulatory effect on CLDN3 expression through its catalytic activity. Furthermore, TET1 plays a role in promoting transcription by targeting demethylation of the CLDN3 promoter region. To examine the region of methylation change in the CLDN3 promoter region, we utilized BSP in TET1KO and TET1OE cell lines. Moreover, we constructed DNA‐targeted demethylation plasmids that specifically target the desired region in conjunction with TET1CD and CRISPR. Following the transfection of cells, the methylation level of the CLDN3 promoter region was quantified, and alterations in CLDN3 mRNA levels were evaluated. The results demonstrated that CLDN3 expression was elevated after demethylation of the F5–F7 (from −16 to 512) region of the CLDN3 promoter. To elucidate the direct correlation between DNA demethylation and transcriptional activation, we examined the effect of CLDN3 promoter methylation on the expression of downstream genes using a dual luciferase reporter assay. The results indicated that the expression of downstream genes was enhanced by a decrease in the methylation level of the F5–F7 (from −16 to 512) segment of the CLDN3 promoter region. Consequently, our findings indicate that TET1 plays a role in regulating CLDN3 expression by targeting the F5–F7 (from −16 to 512) demethylation of the CLDN3 promoter region. The combined results of RNA‐Seq were used to examine the effect of TET1 on EMT in CCA cells. The findings demonstrated that TET1 enhanced cholangiocarcinoma EMT, with this effect dependent on its catalytic activity.

In light of the unconventional role of TET1 in CCA, it is imperative to identify strategies to either inhibit TET1 expression or facilitate TET1 degradation. The literature has demonstrated that post‐translational modifications of proteins significantly affect protein function and stability,^[^ ^38^ ^]^ particularly protein phosphorylation.^[^ ^39^ ^]^ The literature indicates that phosphorylation of TET1 enhances TET1 stability and renders it less susceptible to degradation.^[^ ^17^ ^]^ The hypothesis was that the inhibition of the TET1 proto‐oncogene could be achieved by dephosphorylase‐catalyzed dephosphorylation of TET1, which would weaken its protein stability. We employed HPLC‐MS/MS to detect proteins that may bind to TET1 and then screened for the dephosphorylase PPM1G, which was verified by Co‐IP and GST pull‐down assays, which demonstrated that PPM1G binds to TET1 in the cytoplasm. Immunofluorescence assays indicated that PPM1G likely catalyzes the demethylation of TET1 in the cytoplasm, which results in its inhibition. It is possible that PPM1G catalyzes TET1 demethylation in the cytoplasm and decreases its level. Subsequently, the impact of PPM1G binding on TET1 phosphorylation was investigated. The results demonstrated that TET1 phosphorylation increased with elevated PPM1G expression, while TET1 expression decreased with elevated PPM1G expression. We constructed a D496A enzyme inactivation mutant plasmid of PPM1G for stable transfection of CCA cells. It was found that the loss of dephosphorylated PPM1G also resulted in the loss of TET1 expression regulation. The replication of PPM1G and its mutants in PPM1GKO cell lines also demonstrated that PPM1G relies on its dephosphorylation activity to regulate TET1 expression. A review of the literature revealed that the phosphatases responsible for TET1 phosphorylation are primarily classified into the PKC family and the serine–threonine protein kinase ATM family.^[^ ^17^ ^]^ Consequently, staurosporine (PKCi), which belongs to the PKC‐inhibiting family, and AZD0156 (ATMi), which belongs to the ATM‐inhibiting family, were co‐incubated with cholangiocarcinoma cells. It was found that the phosphatase inhibitors inhibited the expression of TET1, which provides a preliminary experimental basis for using phosphatase inhibitors in treating CCA.

Furthermore, our findings indicate that the regulatory effect of PPM1G on TET1 similarly affects TET1 downstream gene expression and promoter region demethylation. The expression of CLDN3 was dependent on TET1, which PPM1G inversely regulated. In the TET1KO cell lines, PPM1G was unable to affect CLDN3 expression. The detection of methylation levels in the CLDN3 promoter region similarly corroborated the aforementioned conclusion, demonstrating that the dephosphorylation of TET1 by PPM1G resulted in diminished levels of TET1‐catalyzed demethylation of the CLDN3 promoter region. Consequently, we hypothesized that the concurrent administration of phosphatase inhibitors and TET1 inhibitors could impede the EMT in CCA. This hypothesis was subsequently validated by in vivo and in vitro experiments. The combination of phosphatase inhibitors and TET1 inhibitors significantly inhibited the proliferation and migration of CCA cells. The in situ mouse CCA model directly demonstrated that the combination of phosphatase inhibitor and TET1 inhibitor significantly delayed the progression of CCA and prolonged survival. The results of our experiment suggest a new potential avenue for CCA treatment. However, further clinical trials are necessary to confirm the efficacy of this approach.

In conclusion, our experiments demonstrated that PPM1G can inhibit EMT in CCA by catalyzing the dephosphorylation of TET1 and destabilizing it, thereby weakening the targeted demethylation of the CLDN3 promoter region by TET1. Furthermore, we established the PPM1G–TET1–CLDN3 axis, which suggests a new potential pathway for CCA treatment.

## 4 Experimental Section

### Patient Samples

Paraffin‐embedded cancer tissue microarrays were obtained from the National Biochip Engineering Center (Outdo Biotech, Shanghai, China) and approved by the Clinical Research Ethics Committee of Outdo Biotech for use in a series of experiments (Approval number: SHYJS‐CP‐2001002). These included 36 cases of cholangiocarcinoma tissues and 9 cases of normal tissues for which pathology was confirmed. A detailed summary of the clinicopathology of these internal cohorts can be found in Table S1 (Supporting Information).

### Mouse In Situ Cholangiocarcinoma Model

An in situ mouse cholangiocarcinoma model was established using hydrodynamic tail vein injection and the Sleeping Beauty transposon.^[^ ^20^, ^40^ ^]^ The study employed 5 week old male C57BL/6, and tet1‐CKO mice administered a tail vein injection of 2 mL saline. The plasmid mixture consisted of 10 µg mL^−1^ of the pT3EF1aH‐NICD1 plasmid (Addgene86500), 10 µg mL^−1^ of the pT3EF1aH‐myr‐Akt (179909; Addgene), and 0.3 µg mL^−1^ of the transposase plasmid pCMV(CAT)T7‐SB100 (Addgene34879). The pT3EF1aH‐NICD1 and pT3EF1aH‐myr‐Akt plasmids were modified to delete the LoxP fragment before injection into tet1‐CKO mice. The pT3EF1aH‐NICD1 plasmid was a gift from Xin Chen (Addgene plasmid # 86500; http://n2t.net/addgene:86500; RRID:Addgene_129028). The pT3EF1aH‐myr‐Akt plasmid was a gift from Xin Chen (Addgene plasmid # 179909; http://n2t.net/addgene:179909; RRID Addgene_179909). The pCMV(CAT)T7‐SB100 plasmid was a gift from Zsuzsanna Izsvak (Addgene plasmid # 34879; http://n2t.net/addgene:34879; RRID: Addgene_34879). C57BL/6 mice were purchased from Spearfish (Beijing) Biotechnology Co. tet1‐CKO mice were purchased from Cyagen (Suzhou) Biotechnology Co. (C57BL/6J‐Tet1^em1cyagen^, CKOCMP‐52463‐Tet1‐B6J).

### Cell Culture

HEK293T cells were cultured in Dulbecco's modified Eagle medium (DMEM), and TFK‐1 and EGI‐1 cells were cultured in Roswell Park Memorial Institute (RPMI)‐1640, both media being supplemented with 10% fetal bovine serum and penicillin–streptomycin. The cells were then cultured in a humidified atmosphere at 37 °C, 5% CO_2_. The HEK293T, TFK‐1, and EGI‐1 cell lines were obtained from the American Type Culture Collection (ATCC), and all cell lines were confirmed to be mycoplasma‐free before the experiment.

### Plasmid Construction, Transfection, and Lentiviral Packaging

The TET1CD, TET1F1, TET1F2, and PPM1G cDNA were obtained via PCR using total complementary DNA (cDNA) from cholangiocarcinoma cells as a template. The resulting cDNA was then cloned into a phage‐flag or phage‐HA vector to yield TET1CD‐Flag, TET1F1‐Flag, TET1F2‐Flag, and PPM1G‐HA. Point mutant plasmids were generated through PCR using TET1CD‐Flag or PPM1G‐HA plasmids as templates. The human TET1CD and PPM1G cDNA were inserted into the pGEX‐4T1 vector to generate GST‐TET1CD and GST‐PPM1G, respectively. shRNAs of PPM1G (Clone Nos. TRCN0000081209 and TRCN0000001216) were synthesized by BioWorks Biotech (Shanghai, China, 3474) and cloned into the pLKO.1 vector. A comprehensive summary of the primers utilized in this study is provided in Table S2 (Supporting Information). Before utilization, all plasmids were validated through Sanger sequencing. Following the instructions, transfection was conducted using polyethyleneimine (PEI, Yeasen, Shanghai, China). HEK293T cells were co‐transfected with the target plasmids and lentiviral packaging/envelope plasmids (psPAX2 and pMD2.G), and the medium was replaced with a fresh medium after 6 h of transfection. Viral supernatants were collected 72 h after transfection and co‐incubated with target cells in polyglutamine (Yeasen, Shanghai, China) for 8 h before replacing the medium with a fresh medium. Screening with 1 µg mL^−1^ puromycin was performed at least one week after 48 h of infection.

### CRISPR Cas9‐Mediated Gene Editing

To construct TET1 and PPM1G knockdown TFK‐1 and EGI‐1 cell lines, sgRNAs were designed targeting human TET1 or PPM1G and ligated them into the PX459 vector. The TFK‐1 and EGI‐1 cell lines were transfected for 48 h and then screened with 1 µg mL^−1^ puromycin for 3 days. Subsequently, 96‐well plates were inoculated to isolate monoclonal cells. The monoclonal cells were identified through Sanger sequencing and confirmed through western blot analysis.

To construct TET1 overexpressing TFK‐1 and EGI‐1 cell lines, a CRISPRa‐mediated endogenous activation strategy was employed utilizing the lentiSAM v2 (Puro) plasmid, which contained an inactivated Cas9, and the lentiMPH v2 plasmid, which carries a 2A Hygro resistance marker derived from the MS2‐P65‐HSF1 activation helper complex. The lentiSAM v2 (Puro) plasmid was a gift from Adam Karpf (Addgene plasmid # 92062; http://n2t.net/addgene:92062; RRID: Addgene_92062). The lentiMPH v2 plasmid was a gift from Feng Zhang (Addgene plasmid # 89308; http://n2t.net/addgene:89308; RRID: Addgene_89308). The sgRNA was ligated into the lentiSAM v2 (Puro) vector, and lentiviruses were packaged and transfected into cells, which were then screened with 1 µg mL^−1^ puromycin for a minimum of 1 week following the transfection of lentiviruses containing the lentiMPH v2 plasmid. The screening was conducted at a concentration of 1 mm thaumatin for at least 1 week.

The specific sequences of the sgRNAs utilized in this section are provided in Table S2 (Supporting Information).

### CRISPR Cas9‐Mediated Targeted Demethylation

To construct targeted demethylation plasmids targeting different regions of the CLDN3 promoter, two plasmids were utilized: dCas9‐huTET1CD‐T2A‐mCherry, which carries fusion‐inactivated Cas9 as well as TET1CD to target the CLDN3 promoter region and catalyze targeted demethylation, and pSpdCas9‐hudTET1CD‐T2A‐mCherry with TET1CD inactivation for control. T2A‐mCherry was employed as a control for ligating sgRNAs targeting distinct regions of the CLDN3 promoter. pSpdCas9‐hudTET1CD‐T2A‐mCherry (PX458) was obtained from Julia K Polansky (Addgene plasmid # 129027; http://n2t.net/addgene:129027). pSpdCas9‐hudTET1CD‐T2A‐mCherry(PX458) was a gift from Julia K Polansky (Addgene plasmid # 129028; http://n2t.net/addgene:129028; RRID:Addgene_129028).

The specific sequences of the sgRNAs utilized in this section are provided in Table S2 (Supporting Information).

### Bisulfite Sequencing PCR

BSP primers with CLDN3 promoter predictions were designed using the online software MethPrimer (https://urogene.org/methprimer2/). The DNA was extracted using a DNA extraction kit (D3396020000J12T006, Omega), and the BSP transformation reaction was performed using a kit (EM101–02, Vazyme). Subsequently, PCR amplification was conducted using Taq enzyme (C601–02, Vazyme). The PCR products were purified and inserted into the T4 vector (EM101–02, Vazyme). Finally, the colonies were incubated in monoclonal cultures, and ten bacterial clones were randomly selected for sequence determination.

The specific sequences utilized in this section of the BSP are presented in Table S3 (Supporting Information).

### Dual Luciferase Reporter Assay

HEK293T cells were cultivated in 24‐well plates, transfected with a pmirGLO Dual‐Luciferase plasmid that were ligated with a CLDN3 promoter, and the cells were lysed 48 h later with 5 × cell lysis buffer. The resulting supernatant was then collected. The dual‐luciferase reporter assay kit (DL101–01, Vazyme) was utilized to prevent the light from affecting the results. The addition of the luciferase substrate, Renilla luciferase, and stop and reaction buffer was then performed, followed by the transfer of the sample to the Promega luminescence detector, where the firefly luciferase reporter gene activity was measured. After removing the centrifuge tube, the Renilla Luciferase was added and it was placed in the Promega Luminescence analyzer to determine the activity of the kidney luciferase reporter gene.

### Western Blotting

The cell samples were lysed using a RIPA buffer containing a protease inhibitor cocktail and a phosphatase inhibitor. The total proteins were extracted, combined with 5 × loading buffer, and denatured for 15 min at 95 °C. The proteins in each lysate sample were separated by sodium dodecyl sulfate‐polyacrylamide gel electrophoresis and transferred to nitrocellulose membranes (Millipore, USA). Subsequently, the membranes were sealed with 5% Bovine Serum Albumin (BSA) at room temperature and incubated with the primary antibody at 4 °C overnight. Subsequently, the cell membranes were incubated with the secondary antibody, and the signal was observed using enhanced chemiluminescence (Thermo Fisher Scientific). The results were analyzed using Image Lab software (Bio‐Rad). The primary antibodies utilized in this investigation are enumerated in Table S4 (Supporting Information).

### Total RNA Isolation

RNA extraction was conducted using TRIzol reagent (Vazyme, R701) per the instructions. The cells were initially lysed using TRIzol, which was then fully vortexed and shaken with enzyme‐free water. They were cooled and centrifuged at 12000 rpm using a 4 °C centrifuge. Subsequently, the supernatant was aspirated, and an equal volume of isopropanol was added, shaken upside down, and centrifuged at 12 000 rpm in a 4 °C centrifuge. The supernatant should be discarded, and 70% enzyme‐free ethanol should be added to the precipitate. Centrifugation at 7500 rpm at 4 °C should then be performed, after which the supernatant should be discarded and the precipitate air‐dried. Finally, the precipitate should be dissolved in enzyme‐free water to obtain the RNA solution.

### Histology and Immunohistochemistry

H&E staining and immunohistochemistry were conducted with Biossci (Hubei, China). Human or mouse tissues were embedded in paraffin and sectioned at a thickness of 5 µm. The sections were dewaxed, hydrated, and treated with 3% hydrogen peroxide for 30 min to inactivate endogenous peroxidase. The sections were then subjected to staining with H&E or boiling in an ethylenediaminetetraacetic acid (EDTA) antigen repair solution at pH 9.0 for 15 min. Subsequently, the sections were washed with phosphate buffer saline (PBS), blocked with 5% BSA, and incubated with the primary antibody at 4 °C overnight. Following three washes with PBS, the sections were incubated with horseradish peroxidase (HRP)‐conjugated secondary antibody. The color was developed using the DAB kit (Servicebio, G1212).

### Co‐Immunoprecipitation

The cells were lysed with 1 mL of IP lysis buffer (20 mm Tris‐HCl, pH 7.4, 1 mm EDTA, and 150 mm NaCl, and 1% NP‐40) supplemented with a complex protease inhibitor (20138ES05, Yeasen). The lysate was collected and sonicated on ice. Following centrifugation at 12 000 rpm for 15 min, the supernatant was collected, and 50 µL was taken as a whole‐cell lysate. The antibody was added to the remaining supernatant and incubated overnight at 4 °C with protein A/G magnetic beads. The following day, the beads were washed three times with IP lysis buffer and boiled in 2 × upload buffer for 15 min. The beads were then removed by centrifugation, and the precipitated samples were analyzed by immunoblotting.

### GST Pull‐Down Assay

HEK293T cell proteins were purified, and GST fusion proteins were obtained by lysing bacteria with GST lysis buffer (50 mm Tris‐HCl pH 7.4, 0.5% NP‐40, 1 mm EDTA, 150 mm NaCl, and protease inhibitor cocktail). The resulting solution was incubated with GST magnetic beads at 4 °C overnight. The beads were washed three times the following day and boiled in 2 × upload buffer for 15 min before being utilized for immunoblot analysis.

### Immunofluorescence Staining

Cells on coverslips were transfected with TET1CD‐Flag and PPM1G‐HA. Subsequently, the cells were fixed with 4% formaldehyde for 15 min. Following permeabilization of the cell membrane with 0.1% Triton X‐100 for 10 min, the cells were incubated with 5% BSA for 15 min at 37 °C. The primary antibodies were incubated overnight at 4 °C, and the secondary antibodies were incubated the following day at room temperature, protected from light, for 1 h. The cells were washed with PBS, and 4',6‐Diamidino‐2‐phenylindole dihydrochloride (DAPI) was applied for 5 min. Subsequently, the nuclei were stained with DAPI for 5 min. The coverslips were affixed to the slides using an antifluorescence quenching sealer (G1401, Servicebio).

### Cell Proliferation Assay

The viability of the cells was determined using the CCK‐8 (RM02823; Abclonal). A total of 1000 cells were inoculated in each well of a 96‐well plate. Following the instructions, the CCK‐8 reagent was then added when the cells were adhered to the wall of the well. The cells were incubated for 60 min at 37 °C under 5% CO_2_, and the absorbance was subsequently measured at 450 nm. In the clone formation experiments, 1000 cells were planted in 6‐well plates and cultured in 5 mL of complete medium for 4 weeks. The cells were fixed with 4% formaldehyde for 15 min and stained with 1% crystal violet. The number of clones was then counted.

### Transwell Assay

In the course of migration experiments, 2 × 10^4^ cells were enumerated and diluted in 200 µL of serum‐free medium. They were then added to transwells (polycarbonate membrane pore size 8 µm; Corning Incorporated) and placed in 24‐well plates containing 800 µL of complete medium. In the invasion assay, the transwell was preincubated with the matrix gel at 37 °C for 1 h. The transwell was then fixed with 4% formaldehyde for 15 min and stained with 1% crystal violet.

### In Vivo Experiments

The Laboratory Animal Welfare and Ethics Committee of Tongji Hospital, Tongji Medical College, Huazhong University of Science and Technology approved the animal studies (Approval number: TJH‐202201002). A subcutaneous transplantation tumor model was utilized. The mice were 6 weeks old and female BALB/c nude mice from Spearfish (Beijing) Biotechnology Co. Following the experimental design, each mouse was injected with 2 × 10^6^ cells into the right axilla. Tumor growth was monitored biweekly, and mouse weight was recorded. Tumor volume was quantified by measuring the length and width of the xenograft tumors in half. All mice were sacrificed when the most giant grafted tumor reached a diameter of 15 mm. The subcutaneous tumors were excised, photographed, and weighed, and the tumor volume was calculated and fixed in 4% formaldehyde. The liver metastasis model in mice was conducted in the Laboratory Animal Center operating room, where all instruments were sterilized. Following the induction of anesthesia in the mice, a longitudinal incision of ≈8 mm was made in the left posterior axillary line beneath the costal margin to identify and gently extract the spleen. Ligation was performed in the center of the spleen to divide the spleen into two proximal and distal ends with vascular tips. After that, an insulin syringe slowly injected 100 µL of PBS cell suspension containing 2 × 10^5^ representative cells into the proximal spleen. The proximal splenic vessels were ligated, and the proximal splenic vessels and the proximal spleen were severed. Hemostasis was then achieved using an electrocoagulator. Following abdominal closure, the mice were transferred to an incubator and allowed to recover. Once they had regained consciousness, they were relocated to rearing cages. The mice were observed, and their body weights were recorded at 2 day intervals. In the absence of weight gain and observable signs of depression, the mice were euthanized, and their livers were fixed in 4% formaldehyde.

### RNA Sequencing and Enrichment Analysis

RNA samples from the sg‐NC and TET1KO cell lines of extrahepatic cholangiocarcinoma TFK‐1 were subjected to three biological replicates. Total cellular RNA was extracted using the TRIzol RNA extraction kit (Vazyme, R401, Nanjing, China). The quality of the samples was then assessed using an Agilent 2100 Bioanalyzer with the assistance of Novozymes (Beijing, China), and the resulting sequencing data were obtained using an Illumina HiSeq 2500. The sequencing data were subsequently visualized and analyzed using the Integrative Genomics Viewer. The enrichment analysis was based on the principle of hypergeometric distribution, and the clusterProfiler software was used to perform GO(http://www.geneontology.org/), KEGG (http://www.kegg.jp/), and Reactome pathway enrichment analysis on the differential gene sets. All differential genes from each differential comparison set were subjected to enrichment and analysis with the assistance of Novozymes (Beijing, China). In each enrichment catalog, the terms “ALL,” “UP,” and “DOWN” indicate the results of the enrichment analysis for all differential genes, upregulated differential genes, and downregulated differential genes, respectively.

### High‐Performance Liquid Chromatography‐Mass Spectrometry

High‐performance liquid chromatography‐mass spectrometry (HPLC‐MS/MS) sequencing was conducted with the assistance of QL Bio (Beijing, China). HEK293T cells stably transfected with TET1CD‐Flag were immunoprecipitated with an antibody specific for the Flag peptide, while HEK293T cells stably transfected with a control vector were used as a control. The sodium dodecyl sulfate‐polyacrylamide gel electrophoresis (SDS‐PAGE) strips were incubated with Caumas Brilliant Blue staining solution (Dingguo, WB‐0101, Beijing, China) for 30 min, followed by an overnight wash with elution solution (Dingguo, WB‐0111, Beijing, China). Subsequently, additional sample preparation and an online assay were conducted on a QL Bio RIGOL L‐3000 high‐performance liquid chromatography system. The mass spectral raw data were obtained using a Q Exactive HF‐X mass spectrometer with a Nanospray Flex (NSI) ion source. The Homo sapiens sp database and Proteome Discoverer 2.4 software were employed for the search of the library, with the results being evaluated in terms of peptides and proteins at both levels, as well as in terms of each physicochemical property.

### Statistical Analysis

The statistical analysis was conducted using GraphPad Prism 10. Each experiment was performed a minimum of three times. The data were presented as the mean ± standard deviation (SD). The two‐way analysis of variance (ANOVA) or two‐tailed unpaired Student's *t*‐test was employed. The analysis of survival curves was conducted using the Kaplan–Meier method and log‐rank test. *p*‐values less than 0.05 were considered statistically significant, and *p*‐values were expressed as follows. The following *p*‐values were deemed statistically significant: *p < 0.05, **p < 0.01, and ***p < 0.001. Conversely, *p*‐values of 0.05 or above were not statistically significant.

## Conflict of Interest

The authors declare no conflict of interest.

## Author Contributions

W.L. and Y.C. designed the experiments; W.L. and Y.K. performed most of the experiments with the assistance of D.W., J.C., G.W., and Q.W.; W.L. and Y.C. wrote and edited the manuscript; Y.C., B.W., and Y.Q. provided funding support for the project; F.X., W.H., and Y.Q. performed data analysis and supervised the project. All authors read and approved the final paper.

## Supporting information



Supporting Information

## Data Availability

The data that support the findings of this study are available from the corresponding author upon reasonable request.
