# Supplementary material for: PPM1G Inhibits Epithelial–Mesenchymal Transition in Cholangiocarcinoma by Catalyzing TET1 Dephosphorylation for Destabilization to Impair Its Targeted Demethylation of the CLDN3 Promoter
Source: Adv Sci (Weinh). 2024 Oct 30;11(47):2407323. doi: 10.1002/advs.202407323 (PMC11653675; doi:10.1002/advs.202407323)
Supplement: Supplementary file 1 — Supporting Information [file ADVS-11-2407323-s001.docx]

**Supplementary Materials**


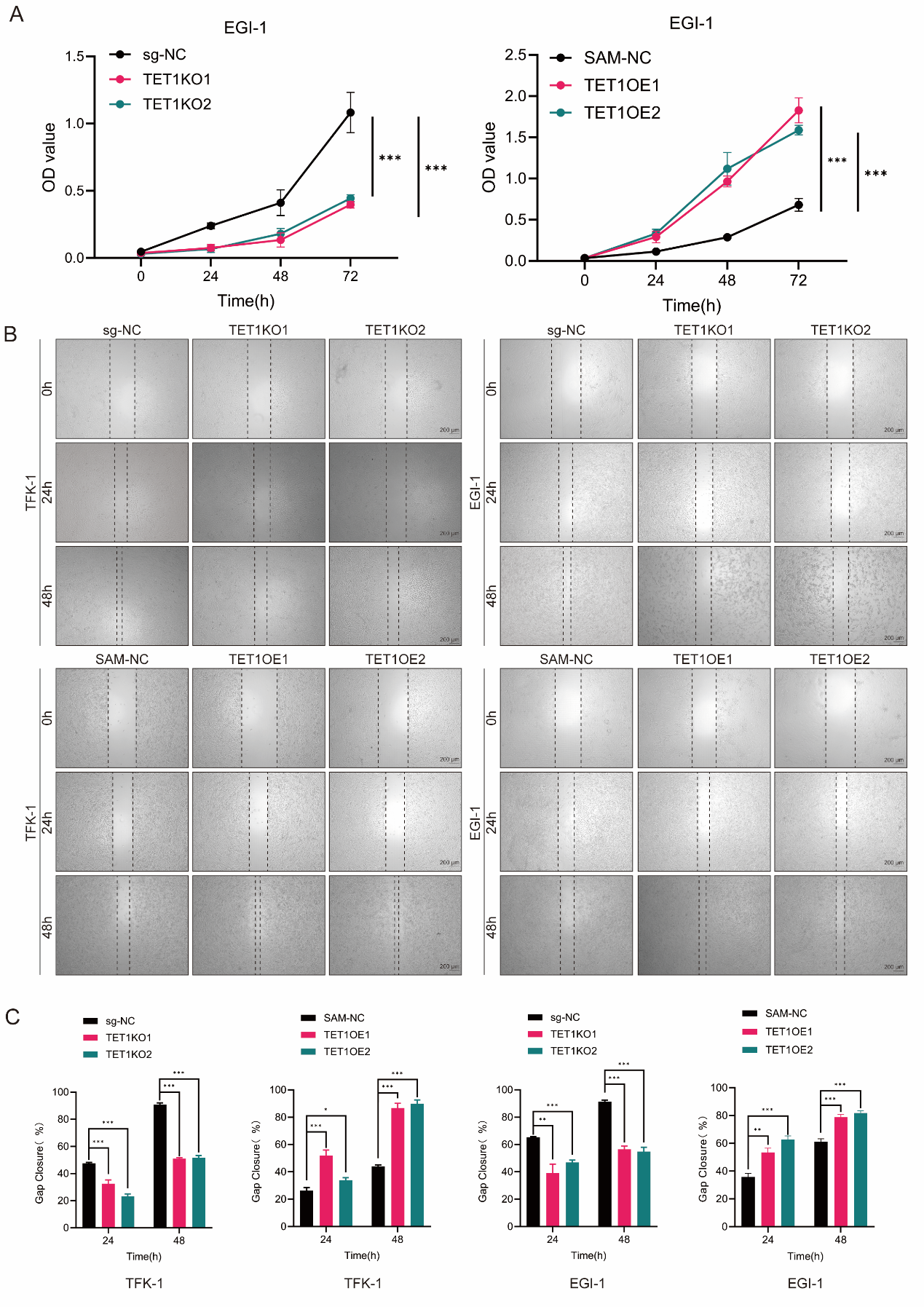


**Figure S1 TET1 promotes cholangiocarcinoma proliferation and migration.** A. CCK-8 assay to detect the proliferation of TET1 knockout and overexpressing EGI-1 cells. B. Wound healing assays to detect TET1 knockout and overexpression of TFK-1 and EGI-1 cell invasion. C. Statistical analysis of migration rates in (B). **P* < 0.05, ***P* < 0.01, ****P* < 0.001, ns, not significant.


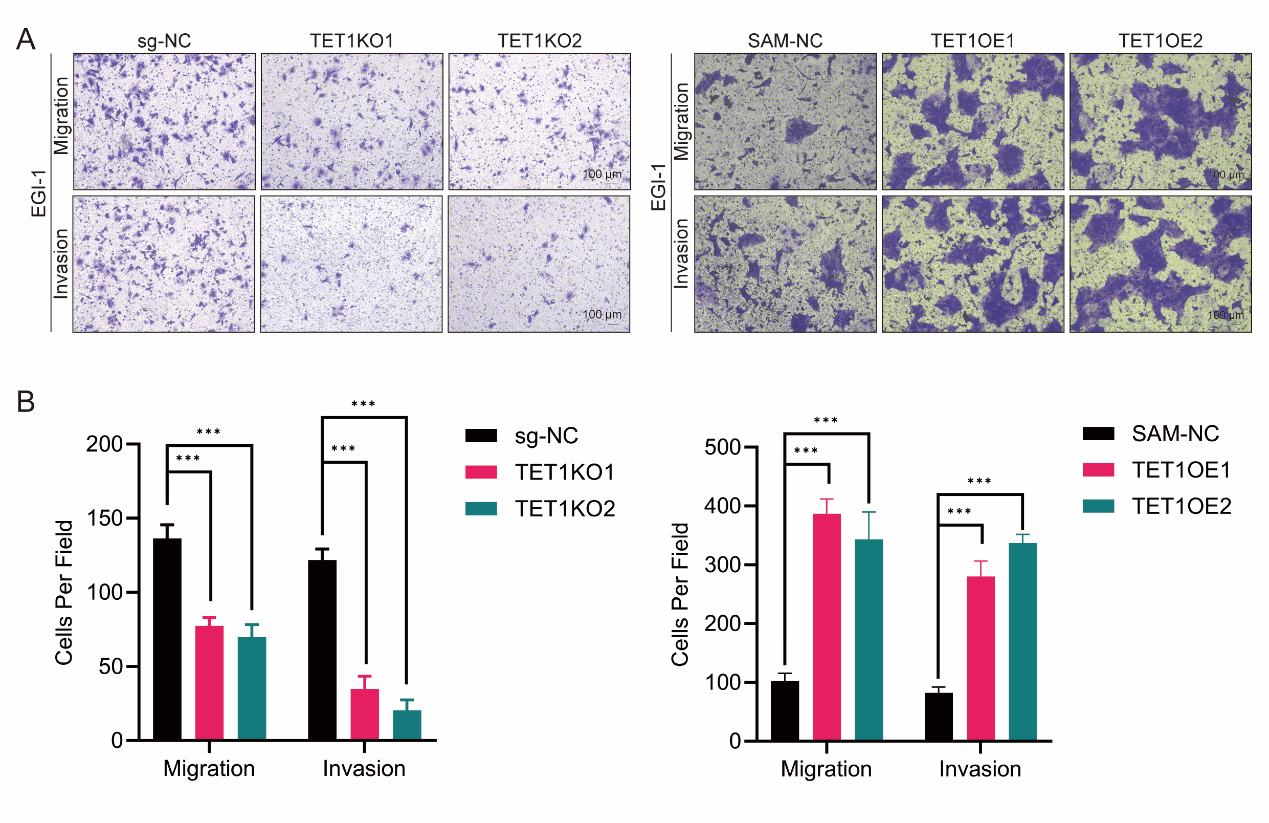


**Figure S2 TET1 promotes cholangiocarcinoma invasion and migration.** A. Transwell assays to detect invasion and migration of TET1 knockout and overexpressing EGI-1 cell lines. B. Statistical analysis of the number of cells per field of view in (A). **P* < 0.05, ***P* < 0.01, ****P* < 0.001, ns, not significant.


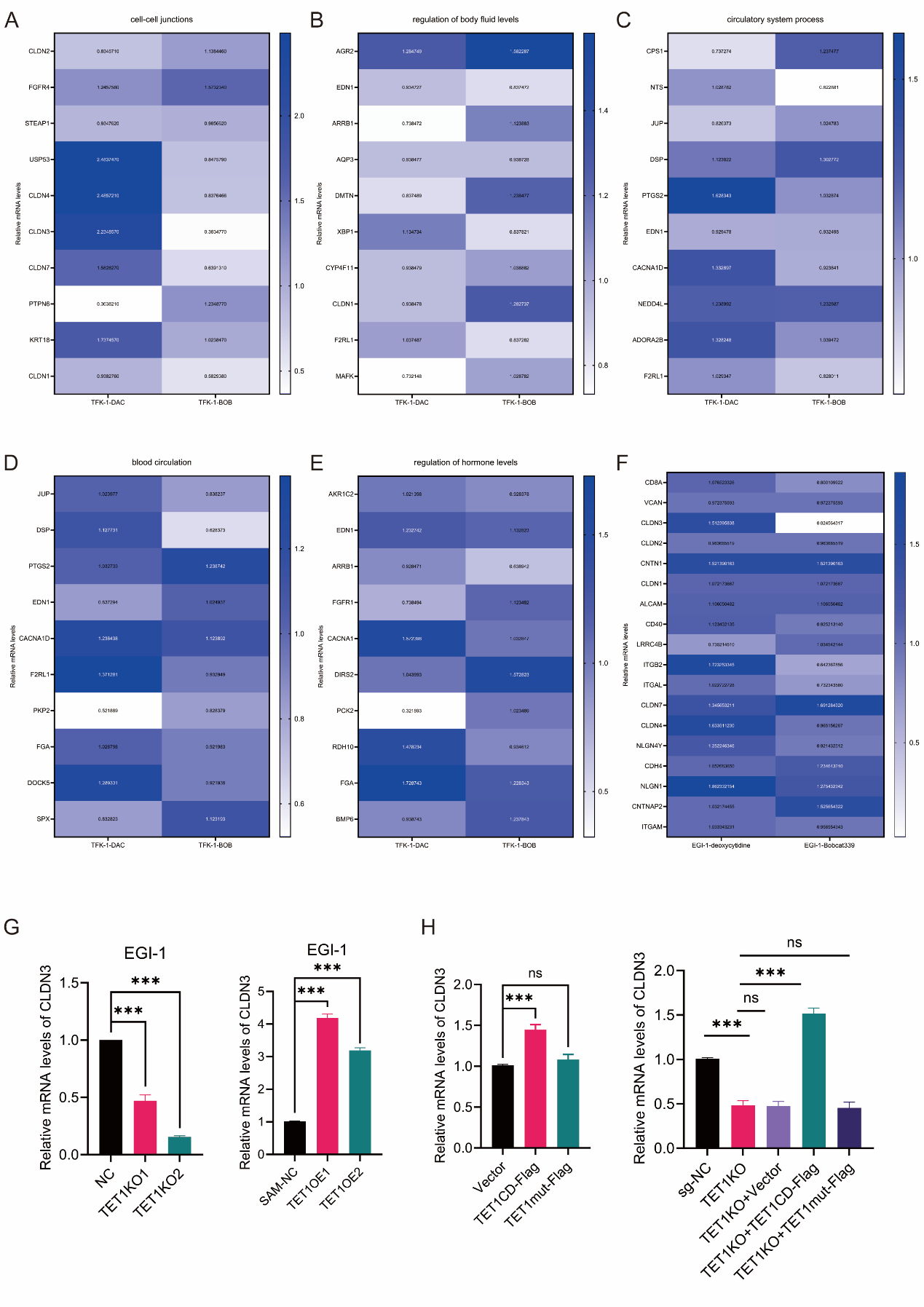


**Figure S3 TET1-dependent catalytic activity promotes CLDN3 expression.** A-E. quantitative real-time PCR (qRT-PCR) was performed to detect the expression of the top ten genes in cell-cell junctions, regulation of body fluid levels, circulatory system process, blood circulation and regulation of hormone levels. F. qRT-PCR assays to examine the changes of gene expression level under the effect of DAC and BOB in EGI-1 cell line. G. TET1 was knocked out and overexpressed in the EGI-1 cell line, and the mRNA level of CLDN3 was detected by qRT-PCR assay. H. TET1CD or TET1mut was overexpressed in the EGI-1 cell line, and CLDN3 mRNA levels were detected by qRT-PCR assay (left). TET1CD or TET1mut was transfected in TET1 knockdown EGI-1 cell lines, and CLDN3 mRNA levels were detected by qRT-PCR assay (right). **P* < 0.05, ***P* < 0.01, ****P* < 0.001, ns, not significant.


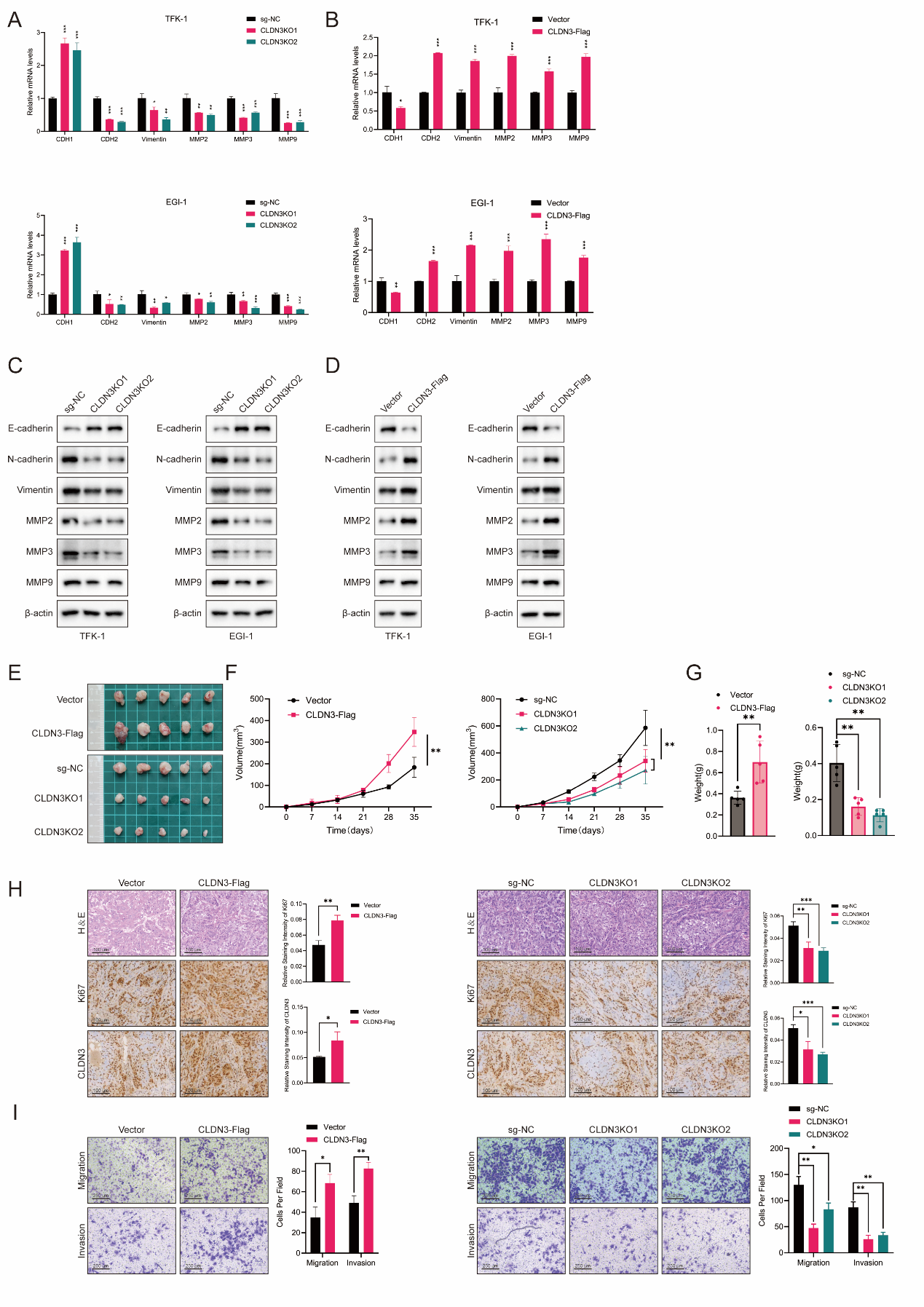


**Figure S4 CLDN3 promotes cholangiocarcinoma proliferation invasion and migration *in vivo* and *in vitro*.** A. qRT-PCR assays were performed to detect changes in mRNA levels of EMT-related indicators after CLDN3 knockout in TFK-1 and EGI-1 cell lines. B. qRT-PCR assays were performed to detect changes in mRNA levels of EMT-related indicators after CLDN3 overexpression in TFK-1 and EGI-1 cell lines. C. WB assays were performed to detect EMT-related indicator protein level changes after CLDN3 knockout in TFK-1 and EGI-1 cell lines. D. WB experiments were performed to detect the changes in protein levels of EMT-related indicators after CLDN3 overexpression in TFK-1 and EGI-1 cell lines. E. Overview of tumors in transplanted xenografts with CLDN3 knockout or overexpression and control cells (n=5). F-G. Volume (F) and weight (G) of tumors in (E). H. H&E staining and Ki67 and CLDN3 staining and scoring of tumors in (E). I. Cell migration assay and Matrigel invasion assay of CLDN3 knockout or overexpression and control cells and statistics of cells per field in (I). **P* < 0.05, ***P* < 0.01, ****P* < 0.001, ns, not significant.


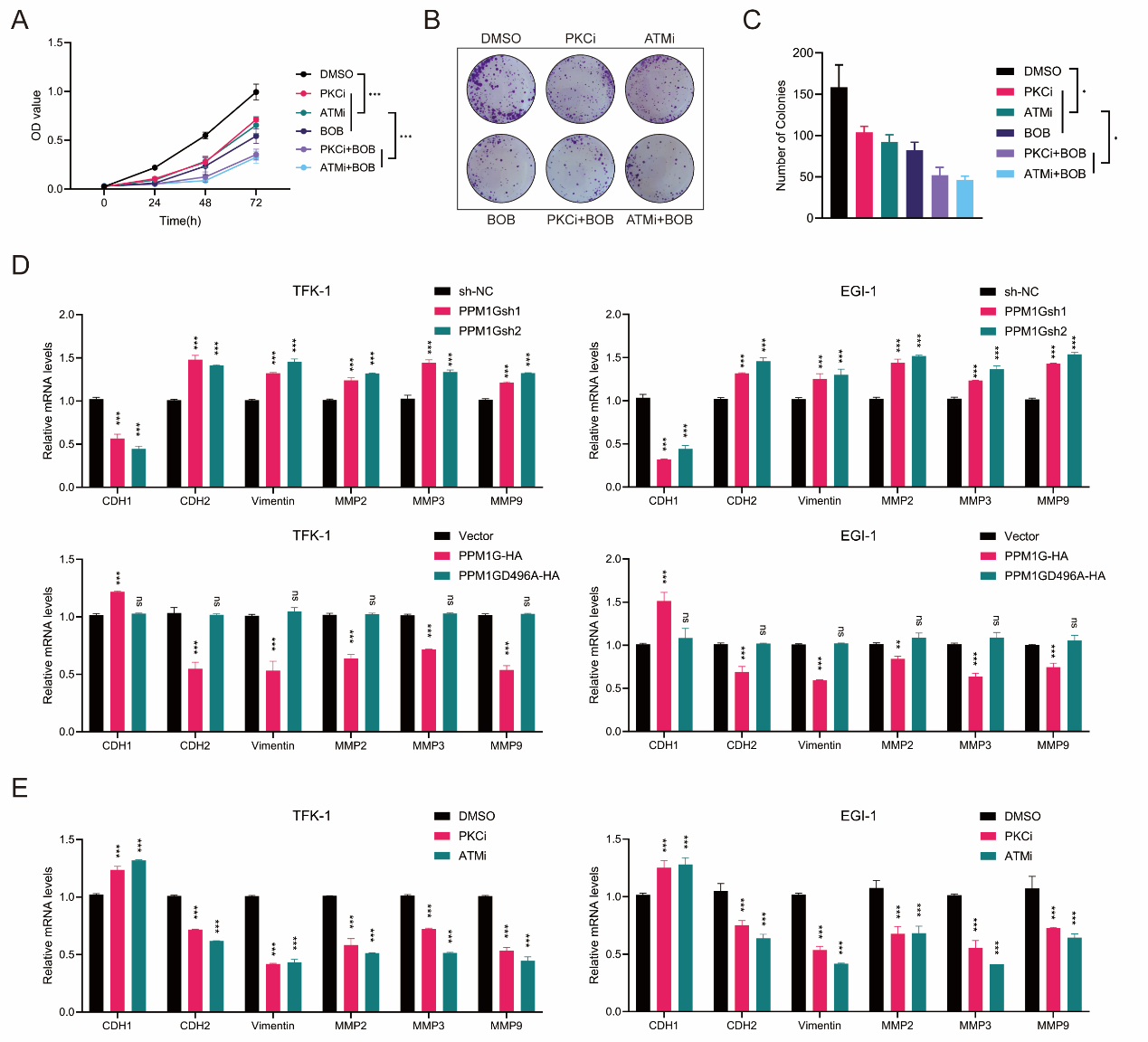


**Figure S5 Combination of a phosphatase inhibitor and a TET1 inhibitor suppresses EMT in CCA.** A. CCK-8 assay was performed to detect the proliferation of EGI-1 cells under the effect of different drugs (DMSO, PKCi, ATMi, BOB, PKCi+BOB, ATMi+BOB). B-C. A clone formation assay was performed to detect the proliferation of EGI-1 cells under the effect of different drugs (DMSO, PKCi, ATMi, BOB, PKCi+BOB, ATMi+BOB) and its statistics. D. qRT-PCR assays were performed to detect the expression of EMT-related mRNA in PPM1G knockdown and overexpressed/mutated TFK-1 and EGI-1 cell lines. E. qRT-PCR assay was performed to detect the expression of EMT-related mRNA in TFK-1 and EGI-1 cell lines under the effect of different drugs. **P* < 0.05, ***P* < 0.01, ****P* < 0.001, ns, not significant.


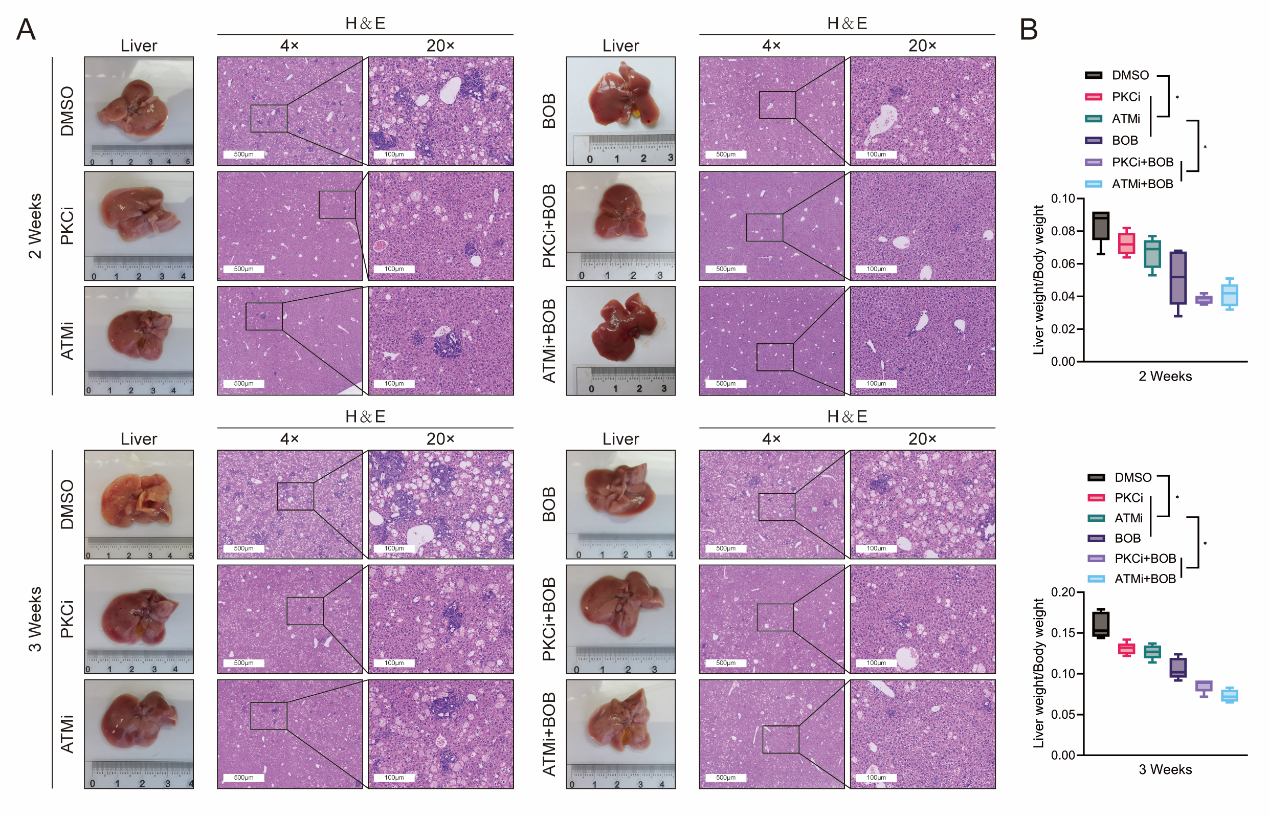


**Figure S6 Phosphatase inhibitor combined with TET1 inhibitor delays cholangiocarcinoma progression in mice.** A. Overview and H&E staining of *in situ* cholangiocarcinoma models in mice under the effect of different drugs. B. Liver weight to body weight ratio in a mouse model of in situ cholangiocarcinoma under the effect of different drugs (n=5). **P* < 0.05, ***P* < 0.01, ****P* < 0.001, ns, not significant.

Table S1 Detailed clinicopathology of tissue microarrays

| **Type of tissue** | **Code** | **Genders** | **Age** | **Pathological Typing** | **Pathological Grade** | **Tumor Location** | **T** | **N** | **M** | **AJCC 7th Edition Clinical Staging** |
| --- | --- | --- | --- | --- | --- | --- | --- | --- | --- | --- |
| Cancer/Paracancer | D18A0005 | M | 71 | Adenocarcinoma | Ⅱ | Inferior of the common bile duct | T2 | N1 | M1 | 4 |
| Cancer/Paracancer | D18A0020 | F | 74 | Adenocarcinoma | Ⅱ | Common bile duct | T2 | N0 | M1 | 4 |
| Cancer/Paracancer | D18A0027 | F | 80 | Adenocarcinoma | Ⅱ | Common bile duct | T1 | N1 | M0 | 2B |
| Cancer/Paracancer | D18A0050 | M | 65 | Adenocarcinoma | Ⅱ | Inferior of the common bile duct | T3 | N0 | M0 | 2A |
| Cancer/Paracancer | D18A0059 | F | 83 | Adenocarcinoma | Ⅱ | Inferior of the common bile duct | T1 | N0 | M0 | 1A |
| Cancer/Paracancer | D18A0067 | F | 58 | Adenocarcinoma | Ⅱ | Bile duct | T3 | N0 | M0 | 2A |
| Cancer/Paracancer | D18A0066 | F | 66 | Adenocarcinoma | Ⅱ | Inferior of the common bile duct |  | N0 | M0 |  |
| Cancer/Paracancer | D18A0104 | F | 61 | Adenocarcinoma | Ⅱ | Inferior of the common bile duct | T3 | N0 | M0 | 2A |
| Cancer/Paracancer | D18A0072 | M | 70 | Adenocarcinoma | Ⅲ | Inferior of the common bile duct | T3 | N0 | M0 | 2A |
| Cancer | D18A0746 | M | 58 | Adenocarcinoma | Ⅰ | Inferior of the bile duct | T3 | N0 | M0 | 2A |
| Cancer | D18A0011 | M | 58 | Adenocarcinoma | Ⅱ | pancreatic section of bile duct | T3 | N0 | M0 | 2A |
| Cancer | D18A0012 | M | 65 | Adenocarcinoma | Ⅱ | Inferior of the common bile duct | T3 | N1 | M0 | 2B |
| Cancer | D18A0014 | M | 51 | Adenocarcinoma | Ⅱ | Middle section of common bile duct |  | N1 | M0 |  |
| Cancer | D18A0016 | F | 60 | Adenocarcinoma | Ⅱ | Common bile duct | T2 | N0 | M0 | 1B |
| Cancer | D18A0033 | M | 57 | Adenocarcinoma | Ⅱ | Inferior of the bile duct | T3 | N0 | M0 | 2A |
| Cancer | D18A0047 | F | 71 | Adenosine squamous carcinoma | Ⅱ | Common bile duct | T1 | N0 | M0 | 1A |
| Cancer | D18A0105 | M | 78 | Adenocarcinoma | Ⅱ | Common bile duct | T1 | N1 | M0 | 2B |
| Cancer | D18A0116 | M | 63 | Adenocarcinoma | Ⅱ | Common bile duct |  | N1 | M0 |  |
| Cancer | D18A0734 | M | 69 | Adenocarcinoma | Ⅱ | Inferior of the common bile duct |  |  | M0 |  |
| Cancer | D18A0738 | F | 48 | Adenocarcinoma | Ⅱ | Common bile duct | T1 | N1 | M0 | 2B |
| Cancer | D18A0035 | M | 51 | Adenocarcinoma | Ⅱ-Ⅲ | Inferior of the common bile duct |  | N1 | M0 |  |
| Cancer | D18A0094 | M | 58 | Adenocarcinoma | Ⅱ-Ⅲ | Common bile duct |  | N0 | M0 |  |
| Cancer | D18A0097 | M | 64 | Adenocarcinoma | Ⅱ-Ⅲ | Common bile duct |  | N0 | M0 |  |
| Cancer | D18A0053 | M | 74 | Adenocarcinoma | Ⅲ | Inferior of the common bile duct | T2 | N1 | M0 | 2B |
| Cancer | D18A0057 | M | 67 | Adenosine squamous carcinoma | Ⅲ | Inferior of the common bile duct | T1 | N0 | M0 | 1A |
| Cancer | D18A0062 | F | 55 | Adenocarcinoma | Ⅲ | Inferior of the common bile duct | T3 | N1 | M0 | 2B |
| Cancer | D18A0078 | M | 60 | Adenocarcinoma | Ⅲ | Common bile duct |  | N1 | M0 |  |

**Table S2 Primer sequences for plasmid construction**

| **Gene Name** |  | **Primer Sequences** |
| --- | --- | --- |
| TET1CD | Forward: | atgtctcgatcccgccatgcaagg |
|  | Reverse: | aactttgggcttcttttccct |
| TET1F1 | Forward: | atgagggaaaagaagcccaaagtt |
|  | Reverse: | agaatctttagttatagacactag |
| TET1F2 | Forward: | gaactgcccacctgcagctgtc |
|  | Reverse: | gacccaatggttatagggccc |
| PPM1G | Forward: | atgggtgcctacctctcccagc |
|  | Reverse: | gtctcgcttggccttcttcttc |
| TET1CD-H1652Y | Forward: | gacttctgtgctcatccctatagggacattcac |
|  | Reverse: | gtgaatgtccctatagggatgagcacagaagtc |
| TET1CD-D1654A | Forward: | gtgctcatccctatagggctattcacaacatga |
|  | Reverse: | tcatgttgtgaatagccctatagggatgagcac |
| PPM1G-D496A | Forward: | gatggtacagggtgtgctaacatgacctg |
|  | Reverse: | caggtcatgttagcacaccctgtaccatc |
| PPM1Gsh1 | Forward: | ccgggccttgtactgtgccaaatatctcgagatatttggcacagtacaaggctttttg |
|  | Reverse: | aattcaaaaagccttgtactgtgccaaatatctcgagatatttggcacagtacaaggc |
| PPM1Gsh2 | Forward: | ccgggaagaggctaccatgactattctcgagaatagtcatggtagcctcttctttttg |
|  | Reverse: | aattcaaaaagaagaggctaccatgactattctcgagaatagtcatggtagcctcttc |
| TET1sg1 | Forward: | ggctcaaaacaagcgaccct |
|  | Reverse: | agggtcgcttgttttgagcc |
| TET1sg2 | Forward: | gttagtgctgactccggtaa |
|  | Reverse: | ttaccggagtcagcactaac |
| CLDN3sg1 | Forward: | cggacggactgactcaccga |
|  | Reverse: | tcggtgagtcagtccgtccg |
| CLDN3sg2 | Forward: | gtgcgcgctccagctcgcgg |
|  | Reverse: | ccgcgagctggagcgcgcac |
| PPM1Gsg1 | Forward: | caccgcaacacggtgaagtgctccg |
|  | Reverse: | aaaccggagcacttcaccgtgttgc |
| PPM1Gsg2 | Forward: | caccgggagaggtaggcacccatgg |
|  | Reverse: | aaacccatgggtgcctacctctccc |
| TET1sg1(SAM) | Forward: | cgagctggatttacccaaac |
|  | Reverse: | gtttgggtaaatccagctcg |
| TET1sg2(SAM) | Forward: | gtctctcgctcaactgtgca |
|  | Reverse: | tgcacagttgagcgagagac |
| CLDN3desg1 | Forward: | caccgctcaggctgagcctgtgcgacgtgtgta |
|  | Reverse: | aaactacacacgtcgcacaggctcagcctgagc |
| CLDN3desg5 | Forward: | caccgccgtcggggcgccgcagctcc |
|  | Reverse: | aaacggagctgcggcgccccgacggc |
| CLDN3desg6 | Forward: | caccggcagccatgtccatgggcctggagatca |
|  | Reverse: | aaactgatctccaggcccatggacatggctgcc |
| CLDN3desg7 | Forward: | caccgctggtgggcgcccagtgcac |
|  | Reverse: | aaacgtgcactgggcgcccaccagc |

**Table S3 Primer sequences for PCR**

| **Gene Name** |  | **Primer Sequences** |
| --- | --- | --- |
| CLDN3BSP1 | Forward: | ctcaggctgagcctgtgcgacgtgtgta |
|  | Reverse: | gcttctctctcctcaccactttggg |
| CLDN3BSP2 | Forward: | cgtgtcaaattctttgggatttg |
|  | Reverse: | cccctcggtccttgtccctctctgct |
| CLDN3BSP3 | Forward: | cgggggctgggtcaggtcccgccctt |
|  | Reverse: | tctccagggcctaaggacagtga |
| CLDN3BSP4 | Forward: | tcactgtccttaggccctggagag |
|  | Reverse: | actgggctggccctgggctggggc |
| CLDN3BSP5 | Forward: | gccccagcccagggccagcccagt |
|  | Reverse: | atggtgcccagccagcccagca |
| CLDN3BSP6 | Forward: | gcagccatgtccatgggcctggagatca |
|  | Reverse: | tcctgcacgcagttggtgcactggg |
| CLDN3BSP7 | Forward: | ctggtgggcgcccagtgcaccaactg |
|  | Reverse: | gtccttgcggtcgtagcctgtgccc |
| β-actin | Forward: | catgtacgttgctatccaggc |
|  | Reverse: | ctccttaatgtcacgcacgat |
| TET1 | Forward: | tcatgggtgtccaattgcta |
|  | Reverse: | gatgagcaccaccatcacag |
| CLDN3 | Forward: | aacaccattatccgggacttct |
|  | Reverse: | gcggagtagacgaccttgg |
| CDH1 | Forward: | cgagagctacacgttcacgg |
|  | Reverse: | gggtgtcgagggaaaaatagg |
| CDH2 | Forward: | agccaaccttaactgaggagt |
|  | Reverse: | ggcaagttgattggagggatg |
| Vimentin | Forward: | gacgccatcaacaccgagtt |
|  | Reverse: | ctttgtcgttggttagctggt |
| MMP2 | Forward: | tacaggatcattggctacacacc |
|  | Reverse: | ggtcacatcgctccagact |
| MMP3 | Forward: | ctggactccgacactctgga |
|  | Reverse: | caggaaaggttctgaagtgacc |
| MMP9 | Forward: | agacctgggcagattccaaac |
|  | Reverse: | cggcaagtcttccgagtagt |
| PPM1G | Forward: | aaggaaggcaagctacagaagg |
|  | Reverse: | cagcagctcttcaatagtcatgg |

**Table S4 A comprehensive account of the antibodies utilized in the study.**

| **Name** | **Code** | **Corporation** |
| --- | --- | --- |
| β-actin | 66009-1-Ig | Proteintech Group |
| α-tubulin | 66031-1-Ig | Proteintech Group |
| TET1 | ab191698 | Abcam |
| CLDN3 | A04393-5 | Boster |
| PPM1G | 15532-1-AP | Proteintech Group |
| Flag-Rabbit | AE121 | ABclonal |
| HA-Rabbit | 51064-2-AP | Proteintech Group |
| Flag-Mouse | 66008-4-Ig | Proteintech Group |
| HA-Mouse | AE008 | ABclonal |
| E-cadherin | BM3903 | Boster |
| N-cadherin | BM3921 | Boster |
| Vimentin | A19607 | ABclonal |
| MMP2 | A19080 | ABclonal |
| MMP7 | A20701 | ABclonal |
| MMP9 | A0289 | ABclonal |
| p-Ser/Phosphoserine | sc-81514 | Santa Cruz |
| Cy5 | GB27303 | Servicebio |
| Alexa Fluor 488 | GB25301 | Servicebio |
| HRP Goat Anti-Rabbit lgG (H+L) | AS014 | ABclonal |
| HRP Goat Anti-Mouse lgG (H+L) | AS003 | ABclonal |
